# Hunting for Novel Routes in Anticancer Drug Discovery: Peptides against Sam-Sam Interactions

**DOI:** 10.3390/ijms231810397

**Published:** 2022-09-08

**Authors:** Flavia Anna Mercurio, Marian Vincenzi, Marilisa Leone

**Affiliations:** Institute of Biostructures and Bioimaging, National Research Council of Italy (IBB-CNR), Via De Amicis 95, 80145 Naples, Italy

**Keywords:** cancer, drug-discovery, Sam domain, EphA2

## Abstract

Among the diverse protein binding modules, Sam (Sterile alpha motif) domains attract attention due to their versatility. They are present in different organisms and play many functions in physiological and pathological processes by binding multiple partners. The EphA2 receptor contains a Sam domain at the C-terminus (EphA2-Sam) that is able to engage protein regulators of receptor stability (including the lipid phosphatase Ship2 and the adaptor Odin). Ship2 and Odin are recruited by EphA2-Sam through heterotypic Sam-Sam interactions. Ship2 decreases EphA2 endocytosis and consequent degradation, producing chiefly pro-oncogenic outcomes in a cellular milieu. Odin, through its Sam domains, contributes to receptor stability by possibly interfering with ubiquitination. As EphA2 is upregulated in many types of tumors, peptide inhibitors of Sam-Sam interactions by hindering receptor stability could function as anticancer therapeutics. This review describes EphA2-Sam and its interactome from a structural and functional perspective. The diverse design strategies that have thus far been employed to obtain peptides targeting EphA2-mediated Sam-Sam interactions are summarized as well. The generated peptides represent good initial lead compounds, but surely many efforts need to be devoted in the close future to improve interaction affinities towards Sam domains and consequently validate their anticancer properties.

## 1. Introduction

Cancer represents one of the major leading causes of death globally, and according to the International Agency for Research on Cancer, the number of new cases is expected to reach ~30.3 millions in 2040 (considering male and female worldwide population, all cancer types, age groups 0–85+). These data support the continuous need for new therapeutic treatments and the identification of novel molecules capable of slowing down the disease severity and related negative outcomes [1,2].

Cancer includes a group of diseases linked to massive uncontrolled cell proliferation in the body. The available strategies to face cancer include surgery, radiotherapy, chemotherapy, and immunotherapy [3]. Surgery works for early or even middle tumors but is associated with different risks (e.g., trauma, bleeding, infection, weakened immunity). Radiotherapy is usually adopted when surgery does not provide benefits to patients, but it is linked to a series of complications and high costs. Chemotherapy relies on the use of chemicals to kill tumor cells [3]. The major disadvantages of chemotherapy are the increasing likelihood of developing drug resistance on prolonged treatment and the consequent recurrence of tumor. In addition, chemotherapeutic agents are generally not able to discriminate normal cells from the cancer ones, thus leading to many side effects. Immunotherapy, acting on the patient’s own immune system, is characterized by fewer side effects compared with chemotherapy and is associated with a longer antitumor effect [3].

### 1.1. Peptides as Anticancer Agents

Peptides represent an interesting class of compounds for building original anticancer tools because they are easier to synthesize and modify than small molecules used in chemotherapy, and they are rather selective compared with the antibodies used in the immunotherapy [3,4]. Nevertheless, peptides can be employed to kill fungi, bacteria, and even tumor cells and also to modulate the immune system. Among the different peptides found in a vast range of organisms, cationic low-molecular-weight peptides have been recognized as anticancer peptides (ACPs) and classified in different manners [3,4]. Indeed, they can be grouped based on structural features (i.e., α-helical, β-pleated sheet, random coil, or cyclic ACPs) or their action (molecularly targeted peptides, “guiding missile” peptides, binding peptides, cell-stimulating peptides) [3,4,5]. Interestingly, the molecularly targeted peptides exert their activities (i.e., cytotoxicity, anti-proliferation, and apoptosis) directly on tumor cells, whereas the guiding missile peptides deliver drugs inside tumor cells [4,5]. Cell-stimulating peptides do not directly act on tumor cells but favor the action of the host immune system against them (i.e., immune system-stimulating peptides) or hamper their proliferation by regulating hormone release via their receptors (i.e., hormone-stimulating peptides) [4,5].

Among the molecularly targeted peptides, AntiMicrobial Peptides (AMPs) have attracted much interest for their involvement in the innate immune systems of several organisms and in the defenses from different bacteria, fungi and viruses [5,6,7]. Antimicrobial peptides can be generally classified as short (i.e., composed of 20–46 residues), basic (i.e., enriched in Lysines and Arginines), or amphipathic [6]. AMPs are considered possible alternatives to antibiotics for treating drug-resistant infections [8]. However, the employment of natural AMPs for therapeutic applications has been limited by diverse issues including poor stability and selectivity and elevated toxicity. In fact, a few clinical trials focused on diverse AMPs failed due to their small efficacy, poorer performance with respect to traditional antibiotics, and safety concerns [9]. In this contest, synthetic AMPs hold great interest as the structural parameters and therapeutic potential of natural peptides can be modulated and improved via chemical modifications suggested by structure–activity relationship studies [8,10]. Cathelicidins represent a family of AMPs identified solely in vertebrates that includes a few members with strong antimicrobial activity towards diverse drug-resistant pathogens [11]. Another feature characterizing the diverse members of cathelicidins family is the wide range of immunomodulatory roles [12]. In 2015 the first cathelicidin from sea snakes (i.e., Hc-CATH) was isolated from *Hydrophis cyanocinctus*. Hc-CATH sequence is made up of 30 amino acids, and its N-terminal portion, which is 19 amino acids long, assumes an α-helical structural organization [13]. Hc-CATH presents strong antimicrobial and anti-inflammatory activities; in fact, in mouse models of lung infection and inflammation, it produces protective outcomes [13]. Nevertheless, the employment of Hc-CATH for therapeutic applications is limited by the rather large dimension and the low stability of the peptide. A series of Hc-CATH derivatives has been designed through amino acid sequence truncation, the amidation of the C-terminal end, and D-amino acid replacements [8]. The smaller antimicrobial peptide HC1-D2 (19 amino acids long) generated through this approach provides large stability towards human serum degradation, presents potent large-spectrum antimicrobial functions with a fast mechanism of action, and displays poor cytotoxicity towards mammalian cells [8].

Another kind of chemical manipulation, that is useful for improving AMPs drug-like features, consists in the introduction of conformational restraints such as intramolecular macrocyclizations through either backbone or side chains [14]. Cyclic AMPs may assume peculiar conformational characteristics and overcome a few peptide limitations such as the poor proteolytic stability and oral bioavailability [15]. Temporins are another class of well-known AMPs that have been initially identified in the amphibian skin secretions of the red frog *Rana temporaria* and represent host defense peptides. Temporins consist of amino acid sequences made up of 10-14 residues with amidation at the C-terminal end. Temporins are cationic peptides with charges ranging from +2 to +3 at physiological pH that are able to assume amphipathic α-helix structures in hydrophobic milieus; these characteristics are responsible for their biological activity [14]. Indeed, temporins contrast the ability of bacteria to acquire resistance by working as membrane-misfolding peptides. Among the roughly 130 identified temporin isoforms, temporin L (TL) attracts attention for its wide-spectrum antimicrobial activity [14]. For example, it is rather potent against Gram-negative bacteria like *Pseudomonas aeruginosa* and *Escherichia coli*. Temporin L is able to inhibit *E. coli* divisome apparatus, interacting with the FtsZ protein and thus hampering its GTPase function [16]. Moreover, synergistic anticancer, immunomodulatory, and antiendotoxin roles have been attributed to TL when used in combination with a few diverse temporins [17]. However, TL also displays substantial hemolytic action at the concentrations needed to produce antimicrobial effects [18]; consequently, it has been employed as a model system to generate synthetic, more bioactive, less toxic derivatives [14]. In detail, to enhance the α-helicity in TL, different stapling techniques relying on several linkers (e.g., lactam, 1,4-substituted [1,2,3]-triazole, hydrocarbon, disulfide) have been exploited, thereby resulting in the assemblage of a library of cyclic TL analogues. The diverse TL peptides have been tested in biological assays to evaluate their antimicrobial, cytotoxic, and antibiofilm activities. Analyses of the peptides consisting of these diverse sets of linkers shed light on the relationship between the enhancement of α-helical content and biological activity. Interestingly, the presence of an olefinic bridge leads to α-helix aggregates, whereas a disulfide bridge correlates with poor helicity and peptides with impaired antimicrobial functions [14].

AMPs can also work against fungi and viruses and function through diverse mechanisms such as disrupting membrane and/or intracellular targets [19]. Lately, the occurrence of fungal infections (i.e., cryptococcosis, candidiasis, and aspergillosis) has greatly increased, causing morbidity and even mortality under immunocompromised conditions including organ transplant, acquired immune deficiency syndrome (AIDS), burn, and cancer [20]. For example, the yeast *Cryptococcus neoformans* represents a human fungal pathogen causing roughly 600,000 deaths every year worldwide [21]. Cationic antimicrobial peptides (CAMPs) can be exploited as model systems for generating synthetic antifungal agents. Binding to the negatively charged microbial surface is favored by the positive charge, whereas an amphipathic secondary structure allows CAMPs to merge with the microbial membranes through the lipid bilayer [22]. Because of their large dimensions, peptides are associated with elevated commercialization costs and consequently possess limited clinical applications. This limitation leads to a certain interest in developing short synthetic peptides that could work as clinically useful drugs [23]. Interestingly, potent antifungal activity has been associated with a few natural CAMPs that are rich in Histidines such as histatins and clavanins and also other CAMPs with elevated Tryptophan content like indolicidin and tritrpticin [24,25]. The heterocyclic Histidine residue appears as a promising candidate to be included in synthetic antifungal peptide motives due to the imidazole side chain, which exhibits buffering capacities under physiological pH conditions and has an amphiphilic character. Instead, the amino acid Tryptophan increases the lipophilicity of synthetic antifungal peptides and contributes to membranes interaction features [23]. A few synthetic peptides made up of a 3-mer amino acid motif including modified Histidine, whose ring has been altered by the insertion of an aromatic moiety at the C-2 position, and by Tryptophan residues show great selectivity and potency against *C. neoformans* [23]. In this contest, an ultrashort amphiphilic peptide “12f” (sequence His(2-biphenyl)-Trp-His(2-biphenyl) and OMe (methoxy) group at the C-terminal side) displays 2-fold improved potency with respect to the clinically employed drug amphotericin B (Amp B) and does not present hemolytic effects when used at minimum inhibitory concentration. Peptide 12f provides good proteolytic stability towards trypsin and is able to function rapidly, similar to Amp B [23]. Moreover, confocal microscopy has demonstrated 12f ability to kill *C. neoformans* cells through the permeabilization of the cell surface, translocation inside the cells, and the induction of genetic material nuclear fragmentation. Further TEM (Transmission Electron Microscopy) analyses have shown that 12f mechanism of action relies on the destruction of the cell wall and plasma membrane of *C. neoformans* cells. If employed in combination with known antifungal drugs (i.e., Amp B and fluconazole), 12f displays an outstanding synergistic effect [23].

UltraShort AntiMicrobial Peptides (USAMPs) attract particular interest in the field of antimicrobial drug development. In particular, conjugated USAMPs are composed of the short amino acid sequence of USAMPs that is likely important for decreasing the toxicity and peptides manufacturing costs [26], whereas the conjugated portion can possibly work as anchoring point providing the needed hydrophobicity to allow binding to bacterial membrane and permeability [27]. Alapropoginine is a conjugated ultrashort hexapeptide whose sequence is composed of alternating subunits of Arginine and Biphenylalanine. The peptide has a proper balance of cationicity and hydrophobicity as Arginine residues provide the positive charge that is necessary for the interaction with negatively charged bacterial membranes, whereas hydrophobic Biphenylalanines are crucial for membrane-disrupting functions. Moreover, in Alapropoginine, the hexapeptide is linked to 2-(6-methoxynaphthalen-2-yl)propanoic acid, which is useful for increasing the antibacterial potency [27]. In fact, Alapropoginine possesses strong antimicrobial activities even against multi-drug-resistant bacteria along with poor toxicity towards human red blood cells. Moreover, when Alapropoginine is employed in combination with conventional antibiotics, it shows a synergistic antimicrobial effect [27].

The conjugation of synthetic or natural AMPs with fatty acids is another interesting route to increasing antimicrobial potency and possibly ensuring antifungal capacity [28,29,30]. LipoPeptides (LiPs) can be generated through the N- or C-terminal acylation of positively charged AMPs with C8–C18 fatty acid tails. The affinity for membranes of this kind of peptides can be modulated by varying the fatty acid tail length, leading to changes in the global hydrophobicity and subsequently in the diverse oligomerization states and general arrangements in solution and membrane environments. Ultrashort Lipopeptides (USLiPs) are usually made up of a peptide portion with 2–4 amino acid residues conjugated to a fatty acid chain (C12–C16) and are provided with positive charge (i.e., +1|+4 range) [29,31]. USLiPs, having amphiphilic character, behave like detergents and can self-associate by forming a hydrophobic core [29]. The capacity of certain antimicrobial LiPs to form aggregates confers additional proteolytic stability and represents an advantage in vivo as the peptide half-life and its efficacy are affected by the ability to resist proteolytic degradation [29]. An interesting work has reported on the design of USLiPs to target *Streptococcus* *agalactiae,* responsible for mastitis in dairy cows [32].

Interestingly, antimicrobial peptides have been identified as promising anticancer agents as well. In fact, tumors cells have outer membranes that are more negative than those found in healthy cells and thus can be selectively attacked by antimicrobial peptides [7,33] with consequent necrosis or apoptosis and eventually cell death [5]. A few examples of such antimicrobial peptides are Pleurocidin and members of its family (extracted from the winter flounder *Pleuronectes americanus*), which exhibit cytotoxic action against human breast cancer cells but not human dermal fibroblasts, and the Buforins family, which are extracted from the stomach of *Bufo bufo gargarizans*, like buforin IIb, active in vitro towards human cervical carcinoma and leukemia cell lines (i.e., HeLa cells and Jurkat cells, respectively) [5].

Instead, “guiding missile” peptides (also known as CPPs, Cell-Penetrating Peptides) play a carrier function through the plasma membrane and can deliver in cells different types of molecules (e.g., DNA, siRNAs, plasmids, oligonucleotides, and proteins) [4,5,34]. CPPs can be grouped considering their physicochemical properties (i.e., cationic, amphipathic and hydrophobic CPPs), their origins (i.e., protein-derived, chimeric and synthetic CPPs), or other clinically relevant features (i.e., cell-specific and non-cell-specific CPPs) [35]. CPPs are able to employ diverse mechanisms to overcome cell membranes [5,34,35,36]. One of them relies on direct penetration, which is energy-independent, mainly characterizes cationic CPPs, and depends on the destabilization of the cell membrane [5,35,36]. Membrane perturbation and direct entry can be related to the peptide-induced formation of inverted micelles in the case of amphipathic peptides or the development of transmembrane potential-modulated water pores; in addition, sphingomyelin and ceramides can also be involved into the direct penetration mechanism by modulating membrane fluidity [34]. Moreover, CPP-mediated cell penetration may exploit different energy-dependent endocytic routes including clathrin- and caveolin-mediated endocytosis as well as macropinocytosis [5,34,35,36]. Endocytic internalization is characterized by the formation of vesicles (named endosomes) in which the peptides are inserted to overcome the cell barrier [34]. Once endosomes are formed and the peptides translocate into cells, different events may occur including the disruption of the endosomal membrane and the formation of transient pores, which allow them to be released and reach their targets in the cytosol [34]. The transactivator of transcription (Tat) peptide from the human immunodeficiency virus (HIV) represents a valuable example of a guiding missile peptide [5,34]. In anticancer therapy, these peptides find several applications, like the so-called Peptide receptor radionuclide therapy (PRRT), by working as carriers for radionuclides (i.e., ^111^In, ^90^Y, or ^177^Lu) [37]. Once these molecules reach carcinoid tumor cells, their radioactive nuclei kill them by emitting radiation [37]. An example is provided by radiolabeled somatostatin analogues, which are made up of three elements: a cyclic octapeptide (“octreotide”), a chelator (e.g., DTPA or DOTA) and a radioactive nucleus [37].

Peptide vaccines represent another intriguing example of cell-stimulating peptides and immunotherapeutic options with applications in anticancer therapy. Peptide cancer vaccines rely on epitope peptides able to stimulate humoral and cellular immune answers through the targeting of tumor-specific antigens or tumor-associated antigens. The interest in peptide vaccines rises from the advantages they are associated with as they are safe, their production is generally easy, and they present the ability to induce antigen-specific antibodies and a T cell answer [37,38]. While enhanced survival and decreased side effects have been associated with a few peptide-based cancer vaccines with respect to canonical therapies, these vaccines in the form of a monotherapy have been insufficient for controlling and curing cancer in the long term [38].

Food is a valuable source of bioactive compounds including peptides, which can have positive influence on human health [6]. Interestingly, different plant-based industrial food products and by-products (e.g., soybean, wheat germ, hemp seeds, rice bran, sesame bran, wheat bran, and rapeseed) are recognized sources of peptides with antioxidant activity, whereas milk and the marine environment are known providers of antimicrobial peptides [6]. In addition, peptides with anticancer activity can be obtained from milk [39]. Indeed, different milk proteins like casein, lactoperoxidase, lactoferrin, α-lactalbumin, and its complexes (i.e., HAMLET (Human Alpha-lactalbumin Made LEthal to Tumor cells), BAMLET (Bovine Alpha-lactalbumin Made LEthal to Tumor cells)), have garnered attention for the ability to block tumor growth, their effects on cancer gene expression, and their cytotoxic activity against certain cancer cells [39]. For example, HAMLET shows anticancer activity against different types of tumors as revealed in studies conducted in animal models of bladder cancer, glioblastomas, and intestinal cancer and on humans (i.e., skin papilloma and bladder cancer) [39].

Organisms from marine environment also constitute a source of promising anticancer peptides [40]. It is possible to isolate marine ACPs from different sources like hydrolysates of proteins derived from coral, fish, and clam, from mollusks, sponges, algae, and fungi [40]. *Callyspongia* is a Red Sea marine sponge from which the Callyptide A peptide can be extracted; it presents recognized inhibitory activity towards the growth of different cancer cell lines (i.e., MDA-MB-231, A549 and HT-29 cells) [41]. An example of anticancer peptide extracted from mollusks is provided by Keenamide A, a cyclic hexapeptide showing a considerable antiproliferative activity against P-388, A-549, and HT-29 cancer cell lines [41]. The advantages associated with the use of marine peptides as anticancer agents with respect to antibodies and proteins are several and include their small size, simplicity to produce, properties favoring passage through cell membranes, low drug-drug interactions, precise targeting, versatility from both chemical and biological points of view, and decreased side effects since they do not settle in the kidney or liver [40].

However, many ACPs show some disadvantages such as high protease sensitivity, short half-life and bioavailability, and defective pharmacokinetics [40]. Therefore, different routes and chemical modifications of the native peptides have been proposed for overcoming these issues [40].

Proteolytic cleavage can be reduced by substituting L-amino acids with D-amino acids [42]. Indeed, the CPPPPEKEKEKEK zwitterionic peptide is an excellent antifouling compound biosensor for α-fetoprotein, a biomarker of several cancers, but its use is hampered by the sensitivity to enzymatic degradation [42]. The substitution of L-amino acids with D-amino acids in the first three positions (i.e., CPP) and in the last three positions (i.e., KEK) significantly increases the stability of the peptide [42].

The strategy proposed instead for enhancing peptide absorption and circulation half-life is based on the employment of nanoparticles (NPs); silica and polymeric NPs are largely used for peptide drug delivery during cancer treatments [43,44,45]. Intriguingly, encapsulation into hollow mesoporous silica nanoparticles (HMSNs) and further coating within a lipid bilayer containing entrapped monophosphoryl lipid A adjuvant enhanced the stability and codelivery efficacy of HGP100_25–33_ and TRP2_180–188_ peptides, thus allowing for the inhibition of tumor growth and lung metastasis in murine melanoma models with a decent safety profile; polymeric NPs based on chitosan instead allow lactoferrin to activate apoptotic pathways in colon cancer and cancer stem cells [43,44,46]. The enhanced bioavailability and stability of peptides can also be achieved by conjugation to polymers or dendrimers [46]. Indeed, the consequent formation of nanoscale self-assemblies leads to an increase in peptide drug size and thus a decrease in renal filtration [46]. For example, cytotoxicity of the KLAK peptide (sequence (KLAKLAK)_2_) towards MCF-7 human breast cancer cells increases for P2-KLAK, the peptide conjugated to a certain poly(β-amino ester) (PAE) that upon self-assembling forms micelle-similar NPs [45]. In fact, P2-KLAK achieves a better internalization by endocytosis, which allows it to efficaciously reach and damage mitochondria and exhibit higher pro-apoptotic activity with respect to the free KLAK peptide [45].

Cyclization is another route to improving ACPs features; it can also be achieved by the reaction of the N-terminal amino and C-terminal carboxyl groups, which thus highly increases the protection of peptides from proteolytic attack by amino and carboxy peptidases [46,47]. As mentioned before when describing AMPs, cyclic peptides can be exploited in a wider number of therapeutic applications due to their higher proteolytic stability in the blood circulation and better tissue penetration and binding properties (i.e., large target specificity and affinity) [47]. In fact, cyclization, by lowering the number of conformations that a peptide can assume in solution, may favor the interaction between peptides and the target binding pockets [46,47]. Diketopiperazines (DKP) represent the simplest form of cyclic peptides that are widely present in nature; those containing a proline residue (i.e., a single proline (P) flanked by a second residue or two prolines) possess the DKP nucleus fused to the pyrrolidine ring and exhibit along with high conformational rigidity and elevated resistance to enzyme degradation higher cell permeability and the ability to strongly interact with various targets [48]. Proline-based DKPs show different properties based on structural features, and they can even find applications in the anticancer drug discovery field. The peptide cyclo (L-Phe-L-Hyp, Hyp = hydroxyproline) is a bicyclic proline-based DKP that has cytotoxic activity against U87 and U251 cancer cell lines; Drimentidine G is a tetracyclic proline-based DKP with promising cytotoxic activity towards HCT-8, Bel-7402, BGC-823, A549 and A2780 cancer cell lines [48].

The identification of novel anticancer peptides can be expensive and time consuming, thus stimulating the development of computational methods of discovering such molecules [49,50,51]. Starting from the observation that ACPs are rich in residues like C, G, I, K, and W that are predominant at several positions within primary sequences, a support vector machine model was set up by exploiting as input amino acid content and binary profiles to search a dataset including experimentally validated anticancer peptides and either random peptides or antimicrobial peptides. In the end, a webserver (http://crdd.osdd.net/raghava/anticp/ (accessed on 2 August 2022)) was set up to predict the minimum number of mutations needed to improve anticancer activity, to discover original ACPs through virtual screening approaches and to recognize ACPs from the analysis of natural proteins [51].

Another approach, “DRACP” (Deep belief network (DBN), random relevance vector machines, AntiCancer Peptide), considers two ACP characteristics (i.e., the sequence and chemical features of amino acid residues) [49]. Concerning the sequence, the average composition of 20 amino acids is analyzed. Regarding the chemical characteristics, amino acids are assembled into six classes based on the patterns of hydrophobic and hydrophilic residues [49].

In summary, this introduction well highlights the potential that peptides hold in the drug development field with particular emphasis on anticancer drug discovery. As a matter of fact, a few peptide-based tools have recently been FDA (Food and Drug Administration) approved for the treatment and diagnosis of different diseases including cancer (Table 1).

### 1.2. Focus of the Review

Given their promising features and potential as anticancer agents, peptides hold a prominent role in drug discovery research. However, peptides, being larger than small molecules, are also ideal candidates as modulators of protein–protein interactions.

However, in order to fight cancer, it is also important to identify novel unexplored protein targets that through their interaction networks regulate crucial signaling pathways related but not limited to cell survival, migration, and apoptosis and whose dysregulation could lead to cancer onset and progression. In this context, the protein-binding modules that mediate protein–protein interactions assume high relevance [61]. Among the protein interaction modules, Sam (Sterile alpha motif) domains represent intriguing examples for their functions in different pathological conditions [62]. Interestingly, the EphA2 receptor plays a crucial controversial role in cancer: it belongs to the Eph family of receptor tyrosine kinases (RTKs) and contains a Sam domain at the C-terminus. Recent studies speculated that peptide inhibitors of heterotypic Sam-Sam interactions mediated by EphA2 could work as anticancer therapeutics [63].

This review will briefly describe Sam domains structural features, functions, and interaction properties. Next, it will report on EphA2-Sam and its interaction partners (i.e., the lipid phosphatase Ship2 (SH2 Domain-containing Inositol Phosphatase 2) and the adaptor protein Odin). In the end, the focus will shift to the different strategies that have been adopted during the last few years in our laboratory to obtain novel anticancer peptides based on the inhibition of Sam-Sam complexes involving EphA2.

## 2. Sam Domains

Since their discovery in 1995, ~65,000 Sam domains have been identified in ~50,000 proteins (data from SMART database [64], http://smart.embl-heidelberg.de/, accessed on 29 July 2022). The name Sam derives from the presence in proteins (i.e., Byr2, Ste11, Ste4, and Ste50) playing an essential role in yeast sexual differentiation and the predicted large α-helical content [45,62,65]. Sam domains are generally small (i.e., with ~70 residues) and possess a globular fold (i.e., five-helix bundle structural arrangement; see Section 2.2.1) with a conserved hydrophobic core [66,67]. Apart from these structural similarities, Sam domains are very versatile as concerning their functions and interaction properties.

### 2.1. The Chameleon Domain

The metazoan reign of eukaryotes represents the main source of Sam domains, but they can also be found in plants, bacteria, and viruses [62,66] (http://smart.embl-heidelberg.de/ (accessed on 29 July 2022) [64]).

The intricacy of diverse organisms seems to be linked to the number of Sam domains they contain. For example, 267 proteins with Sam domains have been found in *Homo sapiens*, whereas 59 in the medium-sized terrestrial mammal *Procavia capensis* (known as rock hyrax) and 29 in *Choloepus hoffmanni* (known as Hoffmann’s two-toed sloth, a type of sloth native of Central and South America) [45] (http://smart.embl-heidelberg.de/ (accessed on 29 July 2022) [64]). Moreover, the presence of Sam domains has been established in all subcellular locations, thus stressing the functional versatility of this protein module [45,66]. Indeed, Sam domains have been associated with different biological functions, such as gene regulation, the modulation of enzyme localization, and a variety of scaffolding properties [61].

Interestingly, the dysregulation of Sam domain functions has been linked as well to different pathological conditions [62]. Mutations in the Sam domains of Bicaudal C homolog 1 (Bicc1) and one of its binding partners (i.e., ANKyrin repeat and Sterile α motif domain-containing six (ANKS6)) lead to kidneys diseases, whereas mutations in the Sam domain belonging to the tyrosine kinase receptor EphA2 (Ephrin receptor A2) are related to cataracts. In addition, Sam domains play a role in cancer onset and progression [62]. The Sam domain of the transcriptional repressor TEL (Translocation-Ets-Leukemia) is involved in hematological malignancies, while the Sam domain of EphA2 receptor is involved in different types of cancer (See Section 3) [62]. Moreover, ectodermal dysplasia is related to mutations in the Sam domain of the p63 protein [62].

This variety of functions, linked to either physiological or pathological conditions, is due to the ability of Sam domains to bind different types of partners, such as proteins that may or may not contain other Sam domains, lipids, and RNA [67]. Mostly, Sam domains can associate through different homo- and heterotypic interactions and even form polymers. EphA2-Sam and the Sam domain of the PI3K (Phosphatidylinositol-3-Kinase) effector protein Arap3 (Arf-GAP with Rho-GAP domain, ANK repeat, and PH domain-containing protein 3) are both able to associate through heterotypic interactions with Ship2-Sam and the first Sam domain of the adaptor protein Odin (Odin-Sam1) assembling in dimers [68,69,70,71], whereas Sam domains from many proteins, including Diacylglycerol Kinase δ1 (DGKδ1), TEL, Bicc1, and tankyrases, are able to generate polymeric arrangements [72,73]. This capacity of Sam domains to self-associate brings positive outcomes to the proteins containing them (e.g., stabilization of the folding, ability to mask hydrophobic surfaces from the hydrophilic environment, and amplified binding capacity by collecting within one multimeric complex a large number of low-affinity binding loci) [66,67].

It is noteworthy that Sam domains are present in almost 40% of ETS (Erytroblast Transformation Specific) family components, which include both transcriptional activators (e.g., ETS-1 and ETS-2) and transcriptional repressors (e.g., TEL and Yan) [67]. These proteins regulate processes like cell growth, differentiation, and embryonic development. In the case of ETS-1 and ETS-2, the role of the Sam domain is to provide a kinase docking site. The Sam domain of ETS-1 contains the amino acid motif (i.e., LXLXXXF, where L = leucine, F = phenylalanine and X = any residue) that is recognized by ERK-2 Mitogen-Activated Protein Kinase (MAPK), which phosphorylates ETS-1 and regulates its function. A similar consensus sequence is responsible for the binding of ETS-2 to Cdk10 (Cyclin-dependent kinase 10), a member of the Cdc2 family kinase, which through phosphorylation, causes the inhibition of ETS-2 transactivation in mammalian cells [67].

DGKδ1 is an enzyme important for modulating the levels of second messengers (such as, diacylglycerol and phosphatidic acid) and subsequently is responsible for the regulation of intracellular signaling [74]. Intriguingly, the activity of DGKδ1 is negatively regulated by Sam-domain self-association, which hampers the translocation of the enzyme towards plasma membrane [74].

Concerning Sam scaffolding properties, an interesting example is provided by CASK (CAlcium/calmodulin-dependent Serine Protein Kinase) and Caskin1, which are two proteins involved in the construction of the presynaptic cytomatrix in the active zones of neural synapses [75]. Caskin1 possesses two central tandem Sam domains that make electrostatic contacts with each other and form a peculiar caskin1 helical polymer where helical turns are generated by the assembly of four Caskin1 tandem Sam modules and eight distinct Sam units. Polymer formation ensures the recognition of the CASK interaction domain (CID) in caskin1, located between the Caskin1 Src homology 3 domain (SH3) and Sam domains, by the Calcium/calModulin-dependent serine protein Kinase domain (CaMK) of CASK [75]. The result is a caskin1 polymer adorned by CASK molecules [75].

The Sam domain of phospholipase KIAA0725p provides an example of lipid interaction module. KIAA0725p in fact binds phosphoinositides through a group of positively charged residues in the Sam domain. Binding to lipids is crucial for the membrane association of KIAA0725p and a mutant, where the positively charged cluster in the Sam domain has been destroyed, results unable of Golgi/ERGIC (Endoplasmic Reticulum Golgi Intermediate Compartment) targeting [76]. Similarly, Sam domains from the α variants of p63 and p73 are capable of binding monosialotetrahexosylganglioside (GM1) and artificial lipid membranes, respectively [62]. The influence of p63-Sam/GM1 interaction on the transcriptional activity of p63α and the important role of p63-Sam/lipid interaction during epidermal morphogenesis have been proposed [77].

Instead, Smaug protein from *Drosophila melanogaster* and its homolog Vts1p from the yeast *Saccharomyces cerevisiae* act as post-transcriptional modulators as their Sam domains bind an RNA hairpin motif named Smaug Recognition Element (SRE) [62,65].

### 2.2. Structural Properties

#### 2.2.1. Sam Fold

**Figure 1:**
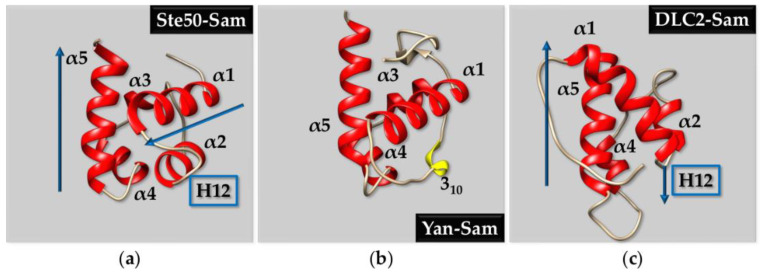
Sam domain fold. (**a**) NMR structure of the *Saccharomyces cerevisiae* Ste50-Sam domain (PDB entry 1Z1V [79]). The α-helices (α1 from V37 to L48, α2 from P55 to E62, α3 from L70 to E72, α4 from L75 to L81 and α5 from L86 to D101) are reported in red. The reciprocal orientation of α5 and the helical H12 hairpin (including α1, α2 and connecting loop) is indicated by blue arrows. (**b**) X-ray structure of the Sam domain mutant A86R of Yan protein (PDB entry 1SV4 [78], chain A). The α-helices (α1 from R56 to F70, α3 from G84 to L89, α4 from R92 to R98 and α5 from G103 to H118) are reported in red, whereas the 3_10_ helix, encompassing residues from F77 to F80, is reported in yellow. (**c**) NMR structure of the DLC2-Sam domain (PDB entry 2H80 [80], conformer n.1 of the NMR ensemble). The α-helices (α1 from Q17 to A30, α2 from P34 to Q38, α3 from I49 to N54 and α4 from V64 to A78) are shown in red. The first 5 N-terminal residues have been removed to better visualize the H12 helical hairpin. The mutual orientation of α5 with respect to the H12 helical hairpin is indicated by blue arrows.

The canonical Sam domain fold includes 5 α helices (α1–α5), with α3 usually being the shortest, organized in a globular five-helix bundle structural arrangement (Figure 1a). [61]. Crystallographic structural studies conducted on a mutant Sam domain from the protein Yan (a transcriptional repressor belonging to the ETS family), in which the A86R mutation allows for a monomeric Sam form, revealed a variant of the five-helix bundle where α2 is substituted by a short 3_10_ helix (Figure 1b) [78].

Known exceptions of the five-helix bundle fold are provided by the Sam domains from tumor suppressors Deleted in Liver Cancer 1 (DLC1) and Deleted in Liver Cancer 2 (DLC2), whose 3D structures are instead made up of a four-helix bundle [61,62]. For instance, in DLC2-Sam, the third α-helix is replaced by an extended secondary structure (Figure 1c) [81]. In addition, a comparison between Ste50-Sam domain (Figure 1a), showing a canonical Sam fold and a DLC2-Sam domain (Figure 1c) proves that while the helical hairpin made up of helices α1 and α2 and connecting loop (named H12 in Figure 1) is usually perpendicular with respect to the last α helix (i.e., α5) in the case of Ste50-Sam, in the uncanonical fold of DLC2-Sam it is oriented almost in an anti-parallel fashion with respect to α5 (Figure 1a,c) [81].

**Figure 2:**
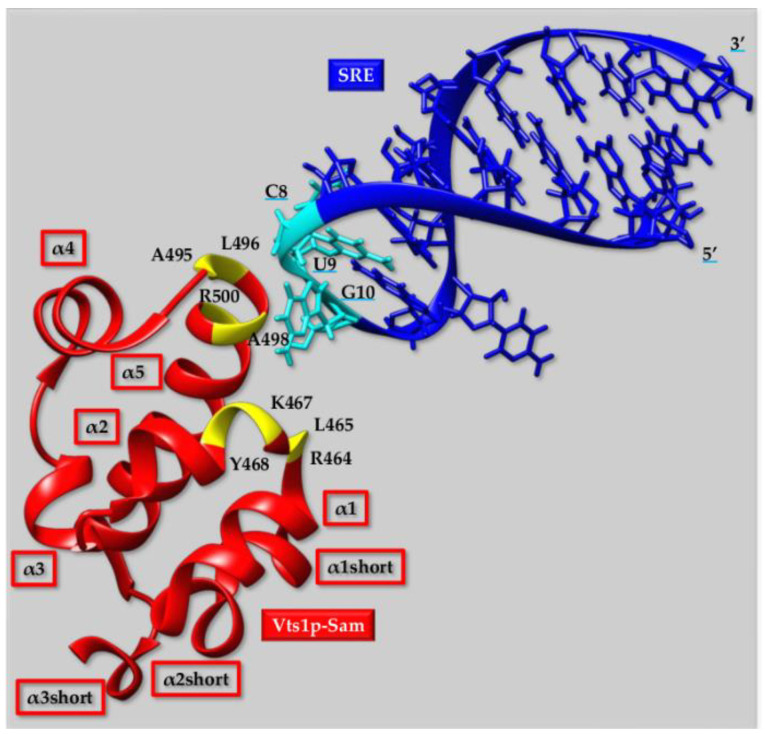
NMR structure of Vts1p-Sam in complex with a 19-nucleotide long SRE RNA (5′-GGAGGCUCUGGCAGCUUUC-3′, PDB entry 2B6G [82]). The Vts1p-Sam domain and SRE are colored red and blue, respectively. The five longer α-helices (α1, α2, α3, α4, and α5) and the three shortest ones (α1short, α2short and α3short) of Vts1p-Sam are labeled. The residues of Vts1p-Sam domain that are involved in RNA binding are highlighted in yellow (i.e., R464, L465, K467, Y468, A495, L496, A498 and R500), whereas the bases of SRE contacting the Sam domain are colored cyan (i.e., C8, U9 and G10).

Another intriguing case is provided by Vts1p-Sam that comprises five α-helices (α1 from I456 to L463, α2 from H466 to L472, α3 from W477 to L480, α4 from D485 to K491, and α5 from L496 to R515) along with three additional shorter helices (i.e., α1short from P444 to L447, α2short P450 to K454 and α3short from R520 to A522) (Figure 2) [82]. As mentioned before this Sam domain is able to interact with the SRE RNA hairpin motif. NMR (Nuclear Magnetic Resonance) studies have revealed close contacts between the guanine G10 from SRE RNA, several Vts1p-Sam hydrophobic residues (L465, A495, L496, A498, Y468), and a basic one (i.e., K467) that are grouped at the intersection between two α-helices, nevertheless, two over five arginines (R464 and R500) in Vtsp1-Sam might also participate in the interaction with RNA according to NMR chemical shift perturbation data (Figure 2). Intermolecular contacts including the ribose rings of cytosine C8 and uracil U9 could be identified too, indicating involvement of the two bases in the binding to Vts1p-Sam (Figure 2) [82].

#### 2.2.2. Sam Domains Self-Association and the Mid-Loop/End-Helix (ML/EH) Model

**Figure 3:**
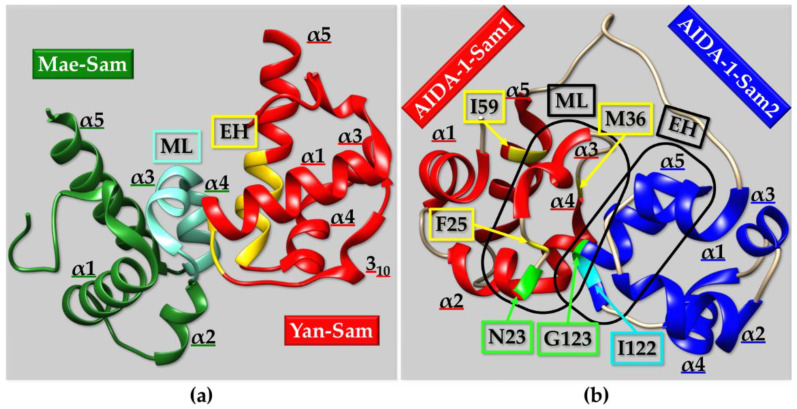
(**a**) X-ray structure of the Yan-Sam (A86R mutant) (red)/Mae-Sam (green) complex (PDB entry 1SV0 [78], chains A and C). The regions defining the EH interface in Yan-Sam are colored yellow, whereas those encompassing the ML interface in Mae-Sam are highlighted in cyan. (**b**) NMR structure of the AIDA-1 tandem Sam1 and Sam2 domains (PDB entry 2KIV [89], conformer n.1). A few crucial residues contributing to the hydrophobic binding surface are indicated in yellow if belonging to Sam1 (i.e., F25, M36 and I59) and in cyan if belonging to Sam2 (i.e., I122). The residues N23 (from Sam1 ML surface) and G123 (from Sam2 EH region), whose backbone _C_O and H_N_, respectively form a characteristic hydrogen bond at the Sam1-Sam2 interface are colored in green.

As described in the previous section, one peculiarity of the Sam domain is its ability to self-associate through homotypic or heterotypic interactions and give rise to oligomers and/or polymers. The most common structural binding topology usually characterizing Sam-Sam interactions is the so called “head-to-tail” or Mid-Loop (ML)/End-Helix (EH) model [83]. The crucial elements of the ML/EH model are the central regions (mostly α3 helix, adjacent helical turns in α2 and/or α4, and relative interhelical loops) of one Sam domain constituting the ML surface and the C-terminal α5 helix and surrounding loops of another Sam unit, forming the EH surface (Figure 3a). “Head-to-head” and “tail-to-tail” topologies are less common [63,84]. A peculiar example is provided by the Sam domain of Sly1; Sly1 is a protein provided with diverse roles such as the modulation of transcription, translation, developmental processes, and cell signaling [85]. Sly-Sam has the typical five-helix bundle fold, but the α2 helix is substituted with a composite helical structure consisting in a 3_10_ helix followed by an α-helical portion. Interestingly, Sly1-Sam self-associates forming a dimer with an unusual structural topology of interaction. Such structural arrangement can be defined as a tail-to-tail like model, with the C-terminal α5 helices facing each other in a parallel manner while, additional contacts are provided by α1 helix, part of helix 2 and residues at the N-terminus [85]. The resulting homodimer shows a stability which is much higher than the one observed in other Sam homodimers. In addition, upon dimerization, the ML region of each monomer remains exposed and thus potentially available to form additional heterotypic ML/EH interactions with EH regions from other proteins [85].

**Figure 4:**
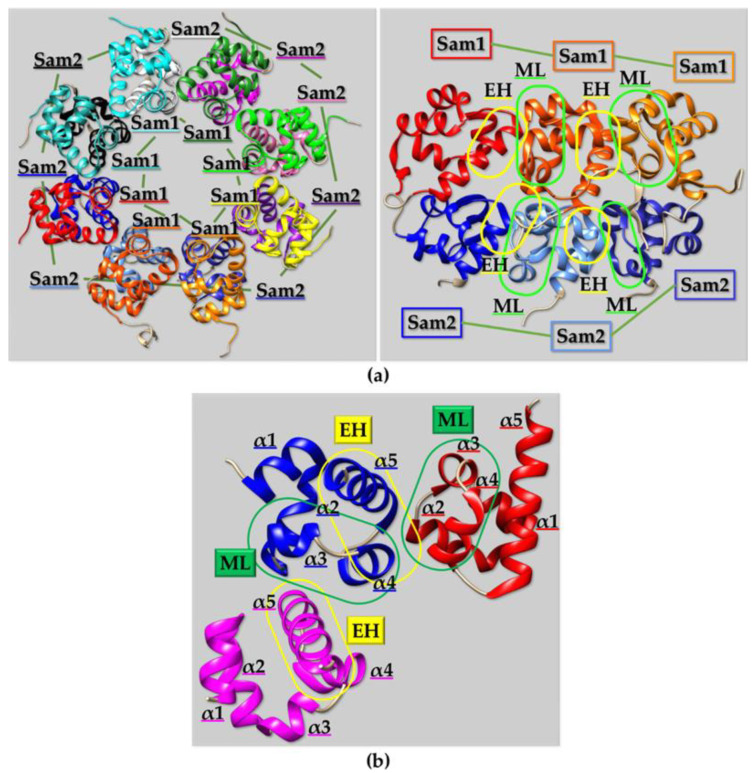
(**a**) Cryo-EM structure of the oligomer formed by the tandem Sam domains of SARM1 (PDB entry 6ZG0 [94]). The structure is devoid of the N-terminal mitochondrial localization peptide and includes the mutation E642Q. Left panel: “UP” view. Red, orange red, orange, yellow, light green, dark green, cyan and light sea green indicate Sam1 domains (residues P404-A477) in single monomers. Blue, cornflower blue, medium blue, purple, hot pink, magenta, white, and black indicate Sam2 domains (residues S485-R543) in each monomer [96]. Right panel: “SIDE” view. Only chains A, B and C are shown. Yellow and light green rectangles highlight EH and ML interfaces. (**b**) Polymeric arrangement formed by Bicc1-Sam domain (R924E mutant) (PDB entry 4RQN [95]). The ML and EH interfaces in each Sam monomeric unit are enclosed in the green and yellow boxes, respectively.

Several ML/EH Sam-Sam homodimers, heterodimers, and polymers have been reported in literature (Figure 3 and Figure 4). Chromosomal translocations that are linked to numerous hematologic malignancies cause fusion of the Sam domain of TEL to an array of proteins, like tyrosine kinases. Cells transformation is induced by activation of the tyrosine kinase domains fused to TEL-Sam upon Sam polymerization. Blocking TEL-Sam polymerization could thus have positive outcomes against hematological malignancies. TEL-Sam polymerizes forming a left-handed helical polymer and studies with mutant TEL-Sam forming small oligomers reveal monomeric units assembling together through the ML/EH model. A dimeric structural arrangement is stabilized by a hydrophobic core at the Sam-Sam interfaces composed of different residues from the ML and EH sites surrounded by several salt-bridges [86]. Yan is another ETS family transcriptional repressor and represents the Drosophila ortholog of TEL. In order to exert its function of transcriptional repressor, Yan needs to polymerize through Sam domain self-association. The phosphorylation and downregulation of Yan, through the receptor tyrosine kinase pathway, is instead facilitated by heterotypic Sam-Sam association with a protein called Mae. Crystallographic studies of the Yan-Sam/Mae-Sam complex indicate that the interface of Yan-Sam responsible for polymerization corresponds to the one recognized by Mae-Sam (Figure 3a). The binding of Mae-Sam to Yan-Sam is much stronger (1000-fold higher) than the homotypic Yan-Sam/Yan-Sam interaction, and thus Mae-Sam by sequestering Yan-Sam causes its depolymerization [78]. This depolymerization mechanism, mediated by heterotypic Sam-Sam interactions, induces transcriptional control. Yan-Sam polymers have a very similar structural arrangement to TEL-Sam polymer [78]. ML and EH surfaces mediate the self-association of Yan-Sam; in addition, Mae-Sam exclusively employs the ML surface to bind the EH surface of Yan-Sam (Figure 3a) [78,87]. Mae-Sam does not possess a functional EH interface and consequently is unable to be inserted into a polymeric arrangement [78]. Yan and Mae Sam domains are indeed involved in different heterotypic Sam-Sam interactions. A MAP kinase (MAPK) called “Rolled” possesses a docking site able to bind the Sam domain from the transcriptional repressor Yan and the activator Pointed-P2 (Pnt-P2), and it stimulates their functions by favoring phosphorylation at precise sites [88]. Interestingly, Pnt-P2-Sam is identified as the target region not only for the docking site of MAPK Rolled but also for the Sam domain belonging to its down-regulator Mae [88]. Mae-Sam and Pnt-P2 bind each other by forming a ML/EH complex where ML and EH regions are provided by Mae-Sam and Pnt-P2-Sam, respectively; hydrophobic residues from the Mae-Sam ML surface and Pnt-P2-Sam EH surface provide the core of the binding interface. Interestingly the EH surface of Pnt-P2-Sam is also responsible for the interaction with MAPK “Rolled”. In this case, the role of Mae appears to inhibit binding of MAPK Rolled to Pnt-P2, thus avoiding phosphorylation and consequently decreasing transcriptional activity. This negative regulation is needed to obtain normal development keeping transcription at the proper level [88].

As introduced above, the self-association of DGKδ1 is mediated by its Sam domain and hampers protein translocation to the plasma membrane [74]. The polymer formed by DGKδ1-Sam domain presents a left-handed helical arrangement (including 6 Sam monomeric units per turn) and the interaction between two adjacent monomers is characterized by the canonical “head-to-tail” topology [74,90]. More in detail, the core of the ML/EH complex is made up of intermolecular contacts provided by three hydrophobic residues in the ML site (i.e., I31, L39 and L47) and by V52 and G53 at the N-terminal region of α5 in the EH site. Additionally in this case, the EH/ML hydrophobic core is surrounded by diverse salt bridges [74]. The defective translocation of DGKδ1 is favored by zinc ions that further stabilize the polymeric arrangement by binding DGKδ1-Sam at multiple sites allowing the sides of polymers to make additional contacts and thus to create highly ordered sheets [90].

The endocytic route, which is mediated by clathrin, requires the action of several adaptor proteins to coordinate the assemblage of clathrin coats and cargo selection. Sla1p is a yeast endocytic adaptor protein that interestingly possesses a SHD2 (Sla Homology Domain 2) domain presenting a Sam fold and a tendency to form a left-handed helical polymer with “head-to-tail” structural topology [91]. SHD2 behaves as negative regulator of endocytosis by hiding a variant clathrin binding motif of Sla1p (i.e., Clathrin Box v(CB): LLDLQ) [91]. In details, intramolecular binding of SHD2 to the vCB motif in the cytosol prevents interaction with free clathrin. Endocytosis starts with assemblage of clathrin-coated vesicles (CCVs), next, Sla1p proteins are engaged by CCVs, where they come into contact with highly concentrated clathrin heavy-chain N-terminal domains, inducing displacement of SHD2 from the vCB motif. Moreover, the increase in the local concentration of SHD2 favors self-association, thus enhancing further exposure of the LLDLQ motif for clathrin binding. During coat disassembly, these Sla1p interactions are reversed, leading to the rebirth of the locked cytosolic state characterized by SHD2 binding to the vCB [55]. This work interestingly establishes a connection between Sam domains and endocytosis.

A few proteins possess two tandem Sam domains that interact with each other by forming ML/EH complexes. This is the case of AIDA-1, which contains two Sam domains—Sam1 and Sam2—interacting in between each other through the ML region (including α2, α3 and α4 in Sam1) and EH region (formed by α5 and adjacent loops in Sam2), respectively (Figure 3b) [89]. The opening of this tandem Sam domain leads to the exposure of a nuclear localization signal and consequently to protein translocation to the nucleus [89]. Three residues from Sam1 domain (i.e., F25, M36 and I59) and two residues from Sam2 domain (i.e., I122 and G123) provide mainly hydrophobic intermolecular interactions within the ML/EH complex (Figure 3b). In addition, the hydrogen bond made up by N23 (on the α2 helix in the ML interface from Sam1) and G123 (at the base of α5 helix in the EH interface from Sam2) is a crucial element for assembling of the ML/EH complex (Figure 3b) [89]. This kind of H-bond appears very important for the first approach between ML and EH sites and is a characteristic of many Sam-Sam “head-to-tail” interactions [92].

Structural studies of the tandem Sam domains (Sam1 and Sam2) of the neuronal scaffolding protein Caskin2 indicate a novel assembling mode that neuronal Sam domains can exploit to combine into large macromolecular organizations in order to enlarge synaptic responses [93]. In this case, the minimal repeating unit is made up by a tandem dimer (composed of four Sam domains Sam1-Sam2/Sam1-Sam2). In detail, Caskin2 Sam tandem composes a domain swapped dimer in which Sam1 associates intermolecularly with Sam2 of a second molecule and the other way around. As every Sam domain within this dimeric arrangement possesses a free binding interface, Caskin2 tandem Sam domains are able to assemble into a branched oligomer, whereas a linear structural topology can be seen in the mammalian homolog Caskin1, which is able to form helical fibers. The intra- and inter-molecular interactions within Sam domains occur according to the ML/EH model. Hydrophobic contacts give a predominant contribution to the head-to-tail interaction model, although ionic contacts appear important either at the intramolecular Sam-Sam binding surface of the dimer and at the intermolecular level [93].

Another intriguing example is given by the Sterile Alpha and toll/interleukin-1 Receptor Motif-containing protein 1 (SARM1). SARM1 plays a crucial role in regulating programmed axonal degeneration and it is thus considered a putative therapeutic target. SARM1 contains two Sam domains in tandem (Sam1 and Sam2), both SARM1 and the isolated tandem Sam1-Sam2 domains give rise to octamers in solution (Figure 4a) [94]. The octameric organization of SARM1 is made up of a first inner ring along with a second external peripheral one. Sam1 and Sam2 constitute the inner ring as eight Sam1-Sam2 units assemble in between each other to generate the internal skeleton. Sam1 and Sam2 present analogous surfaces in terms of charge and hydrophobicity distributions. Indeed, ML surfaces appear negatively charged while EH surfaces are positively charged. Interestingly, on the head of the α5 helices, in the EH regions, hydrophobic residues (i.e., I461 from Sam1 and V533 from Sam2) enter into complementary hydrophobic pockets positioned at the midpoints of the mutual ML interfaces. Thus, electrostatic contacts engage primarily adjacent Sam1-Sam2 units and next, the hydrophobic interactions stabilize the structural arrangement [94]. The tandem Sam1-Sam1 and Sam2-Sam2 lateral contacts allow gaining a very strong interaction and are responsible for the uncommon closed ring structural topology. In fact, as described before, if only one Sam domain is engaged into lateral EH to ML contacts, a helical polymeric arrangement is generated like that observed for Bicc1-Sam (Figure 4b) [95].

## 3. The EphA2 Receptor and Its Controversial Role in Cancer

**Figure 5:**
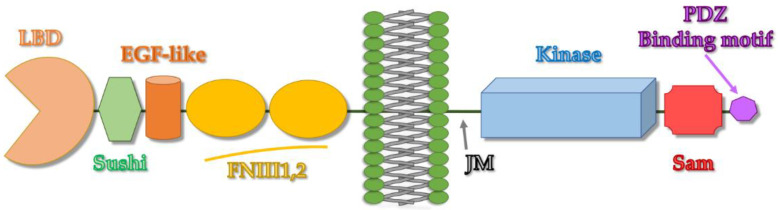
Eph receptors modular domain organization. In detail, the extracellular region of Eph receptors is positioned at the N-terminal side and includes a ligand binding domain (LBD), a Sushi domain, a cysteine-rich domain with an epidermal growth factor (EGF)-like motif, and two fibronectin-kind III repeats (FN III1 and FN III2). A unique transmembrane domain (TM) precedes the intracellular C-terminus including first a juxtamembrane region (JM) followed by a kinase domain (KD), a sterile α motif (Sam), and a PDZ interaction sequence [103].

The biggest subfamily of RTKs is represented by the Eph (Erythropoietin-producing hepatocellular) receptors that interact with membrane-bound ligands called ephrins [97,98]. EphA1 was the first discovered Eph receptor tyrosine kinase and was cloned in 1987 from a hepatocellular carcinoma (HCC) cell line; next, in 1990 the screening of a cDNA library of HeLa cells brought the identification of EphA2 [99,100,101,102]. Currently, 14 Eph receptors and 8 ephrin ligands are known, there are nine Eph receptors of type-A (EphA1-8, and EphA10) and 5 Eph receptors of type-B (EphB1-4,6). Ephrins are classified into two groups based on structural features: ephrin-A (including ephrin-A1-6) and ephrin-B (comprising ephrin-B1-3) [100]. Eph receptors represent single transmembrane proteins containing extra- and intracellular domains where interactions with different ligands and inherent enzymatic activities take place (Figure 5) [97].

EphA receptors usually bind ephrin type-A ligands, which employ a glycosylphosphatidylinositol (GPI) linkage to stay attached to the cell membrane. EphB receptors commonly bind ephrin type-B ligands, which comprise a transmembrane-crossing domain along with an intracellular portion including a cytoplasmic segment and a PDZ (Postsynaptic Density protein 95, discs large 1, and Zonula occludens-1) interaction motif [98,104]. Binding between ephrins and the Eph receptors may induce both “forward” and “reverse” signaling propagating in Eph receptors and ephrins expressing cells, respectively [99,105,106]. The canonical Eph/ephrin pathway is triggered following the interaction of the ligand positioned on one cell to the receptor located in trans on an adjacent cell [107,108]. This interaction leads to a conformational change affecting receptor and ligand that permits oligomerization with near Eph/ephrin complexes and ultimately leads to autophosphorylation of conserved tyrosine residues positioned in the juxtamembrane region [102]. Following these events, the kinase domain becomes exposed and consequently is activated thus starting a series of phosphorylation events affecting the intracellular region that trigger engagement of downstream effector proteins provided with SRC homology 2 (SH2) domains [103,109]. In detail, the activated receptors may interact with a few proteins, such as the guanine-nucleotide exchange factor Vav, ephexin, the Src family tyrosine kinases, focal adhesion kinase (FAK), and the p85 subunit of phosphatidylinositol 3-kinase (PI3K) for modulating the pathway. Conversely, ephrins orchestrate reverse signaling via their interaction with transmembrane proteins, like the neurotrophin receptor p75NTR, TrkB (Tropomyosin receptor kinase B) and Ret (Rearranged during transfection) receptor [99]. Eph receptor signaling is involved in several biological mechanisms, mainly cell–cell adhesion and repulsion. Hence, Eph receptors and ephrins play pivotal roles in the development of blood vessels in the embryo and in neuronal targeting, as well as in the patterning of tissues. EphA2 normal functions are also correlated to the development of embryonic lens and kidney, mammary epithelial branching morphogenesis, and bone homeostasis [106]. In addition to these roles, EphA2 is a key regulator of tumorigenesis and cancer progression [103]. In fact, EphA2 is overexpressed in many solid tumors, like gastric, esophageal, colorectal, cervical, breast, ovary, prostate, pancreas, neck, renal, lung, bladder cancers, melanoma, and glioblastoma [110,111,112,113,114]. This EphA2 overexpression is associated with tumor metastasis and poor patient survival and consequently linked to a highly aggressive cancer phenotype [105,114,115]. Certain studies have also indicated that EphA2 favors the Epithelial–Mesenchymal Transition (EMT) and contributes to the preservation of cancer stem cell-like characteristics [116,117]. Intriguingly, mice that are EphA2-deficient, or cancer cells with EphA2 knockdown, exhibited reduced tumor development, angiogenesis, and metastasis [99]. The role of EphA2 in cancer is controversial as opposite outcomes, linked to malignant transformation and progression, are induced by the ligand-dependent signaling (i.e., canonical pathway) respect to the ligand-independent signaling (i.e., non-canonical pathway) [103]. Eph receptors non-canonical pathway represents receptor signaling in the absence of ligand interaction and activation of kinase activity, and occurs through cross-talk with diverse surface receptors and binding to intracellular kinases [103]. It has in fact been reported that EphA2 can form heterodimers with HER2 (Human Epidermal growth factor Receptor 2), EGFR (Epidermal Growth Factor Receptor), E-Cadherin, and integrins and modify downstream signaling through a ligand-independent and non-canonical route [103]. Importantly, it has also been described that EphA2 may be activated by phosphorylation at serine 897, located in the linker region between kinase and Sam domain, an event regulated by kinases like AKT, RSK, and PKA [103,115,118,119]. The diametrically contradictory functions of EphA2 in cancer (i.e., pro- and anti-oncogenic roles) have been linked to diverse modulation of cell migration and invasion operated by either the canonical or non-canonical signaling [115]. Although activation of EphA2 following ephrin-A1 ligand interaction blocks chemotactic migration of glioma and prostate cancer cells, EphA2 upregulation induces cell migration through a ligand-independent mechanism. Unpredictably, enhanced migration through the ligand independent route may be linked to phosphorylation of EphA2 on S897 by AKT. Mutation of S897 to alanine blocks cell motility induced by the ligand-independent pathway. Intriguingly, following EphA2 stimulation with the ephrin-A1 ligand, activation of AKT by growth factors is annulled and dephosphorylation on S897 in EphA2 occurs [115]. It has been reported as well that the levels of several EphA2 ligands, such as ephrin-A1, are highly decreased in different types of aggressive tumor cells, mainly in those characterized by EphA2 upregulation. This observation, along with the above-reported data, indicates that the EphA2 canonical tyrosine kinase-based signal pathway works mostly as an antioncogenic route, whereas the non-canonical tyrosine kinase-independent pathway should play a crucial role in tumor malignancy [99]. Indeed, as tumor progresses, EphA2 receptor can acquire pro-oncogenic ligand-independent roles [120]. On the contrary, these outcomes can be inverted following ligand stimulation leading to EphA2 anti-oncogenic signaling [120].

Phosphorylation of S897 in human astrocytoma (i.e., a type of brain cancer) has been related to AKT activation and tumor grades and let speculate that the AKT-EphA2 crosstalk may play a crucial role in brain tumor evolution [115]. The role of EphA2 in GBM (GlioBlastoma Multiforme) has been widely investigated. Studies of GBM cell lines and collective biopsies, have revealed enhanced expression of EphA2 protein as well as mRNA in these samples compared with healthy brain tissues [120]. Deregulation of EphA2 receptor levels has also been evidenced in prostate cancer and connected to tumor growth. EphA2 expression levels in several prostate cancer cell lines and patient-derived samples have been analyzed and evidenced that metastatic prostate cancer cells present EphA2 protein expression levels 10–100 fold higher than those in non-invasive prostatic epithelial cells [120]. Moreover, eighty-nine patient derived ovarian tumors and four ovarian cancer cell lines have been analyzed and results point out that EphA2 expression is linked to aggressive characteristics of ovarian carcinoma [120]. EphA2 has also been related to breast cancer EMT and metastasis following elevated extracellular matrix rigidness; the process has been related to ligand-independent pathway and also occurs with engagement of LYN (Lck/Yes Novel) tyrosine kinase to S897-phosphorylated EphA2 [103,121]. Several pro-oncogenic roles of EphA2 are related to the ligand-independent pathway in lung cancer. Certain studies show that either treatment with ephrin-A1-Fc or EphA2 knockdown in NSCLC (Non-Small Cell Lung Cancer) cells lowers cell proliferation and migration in vitro mainly through reduced S897 phosphorylation and downregulation of ERK1/2 (Extracellular signal-Regulated Kinase 1/2) activity [103,122,123,124]. Non-canonical EphA2 pathway has also been linked to several features of melanoma evolution (i.e., migration and invasion, metastasis, and drug resistance) [103,125].

Based on the different cellular contexts, mutual exclusion of EphA2 canonical and non-canonical pathways can occur or the two pathways may take place at the same time and/or possibly they can cross-talk by regulating each other. A recent work by Lechtenberg et al. [126] shows that not only S897 but multiple Serine and Threonine residues in the kinase-Sam linker region of EphA2 (i.e., ^892^SIRLPSTSGS^901^) can be phosphorylated [126]. The analysis of mass spectrometry data associated with diverse non-small cell lung cancer and breast cancer cell lines revealed a robust connection between S897 phosphorylation and the phosphorylation of other residues such as T898, S899, and S901, whereas poor linkage has been established with S892 phosphorylation. Structural data have been collected on EphA2 mutant constructs containing mutations in the kinase-Sam linker, where S and T amino acids have been replaced by glutamic acids to simulate phosphorylation [126]. A more closed conformation of the intracellular EphA2 domains is observed if the linker region is not phosphorylated, as electrostatic interactions occur in between positively charged surface of the Sam domain and negatively charged surface of the kinase domain. Conversely, the accumulation of negative charges that simulate linker phosphorylation leads to a kind of open conformational state characterized by reduced contacts between kinase and Sam domains. It appears like phosphorylation of S897 is able to modulate EphA2 conformation in the intracellular region not in the context of a single residue but enhancing phosphorylation of additional amino acids in the kinase-Sam linker [126]. Several kinases have been identified as capable of inducing in vitro phosphorylation of diverse serine/threonine residues in the EphA2 linker. However, the same study indicates that an EphA2 5Glu mutant can form larger oligomers upon stimulation with ephrinA1-Fc thus pointing out that EphA2 adjacent interactions on the cell surface are modulated by linker phosphorylation [126].

### 3.1. Possible Therapeutic Approaches

Due to the correlation of aggressive pathological and clinical characteristics in human cancers and EphA2 upregulation, many studies have been focused on the possibility to treat cancer through EphA2 downregulation. For example, EphA2 in cancer cells has been silenced through small interfering RNAs (siRNAs), representing intriguing tools for gene knockdown [97,127]. If short hairpin RNAs are employed to induce specifically depletion of EphA2 in melanoma cells, where the receptor is upregulated, a large reduction of cellular viability, colony formation and migration in vitro is encountered along with a significant loss of tumorigenic capacity in vivo [128]. Suppression of EphA2 expression in cells derived from pancreatic adenocarcinoma, through sequence-specific siRNA, leads to delayed tumor growth in a mouse xenograft model [129]. Moreover, stimulation with EphA2-targeting siRNA highly decreases malignancy in glioma, breast cancer cells and non-small cell lung cancer (NSCLC) [130,131,132]. However, despite the great success in in vitro knockdown, in vivo siRNA delivery is challenging [97,133].

Nevertheless, antibodies (Abs) and ad hoc designed synthetic ligands (peptides and small molecules) binding EphA2 could inhibit signaling by favoring receptor endocytosis (i.e., internalization and consequent degradation) [97]. To date various Abs targeting EphA2 are being evaluated in pre-clinical or clinical trials. EA5 represents a mouse mAb (monoclonal Antibody) that targets the LBD of EphA2 and simulates ephrin ligand binding [134,135]. Thus, EA5 works as an agonist that inhibits EphA2 ligand-independent signaling and causes EphA2 phosphorylation and degradation. EA5 induces reduction of EphA2 expression in vitro, and treatment with this mAb also blocks the growth of several tumor cell lines [135]. Similarly, another mAb also able to interact with EphA2 LBD, known as MEDI-547 or 1C1, has been identified through phage display technique [120]. Thus, 1C1 possesses agonist actions and causes fast tyrosine phosphorylation, internalization and consequent degradation of EphA2 [120,136]. By inducing receptor endocytosis, 1C1 can be employed to deliver cytotoxic agents to tumor cells overexpressing EphA2 [137].

Peptides are compounds that can be also implemented for targeting selectively EphA2 receptor [138,139]. Two 12-mer ephrin mimetic peptides (i.e., YSA and SWL) were discovered through selection of a phage display peptide library focused on EphA2 [140]. The YSA and SWL peptides target EphA2 LBD by working in an agonistic manner, and they are able to compete not only with each other but also with ephrinA1 for the interaction with EphA2 [140]. YSA and SWL cause EphA2 receptor activation and block AKT and ERK1/2 MAP kinases phosphorylation in endothelial cells [140,141].YSA and SWL peptides can be also employed as drug delivery tools [139], for example if linked to nanogels that are loaded with siRNA, or diverse drugs including chemo-sensitizing, and radio-sensitizing agents [120]. Indeed, different synthetic routes have been used to combine the YSA peptide to anti-cancer and/or imaging agents. The YSA peptide has been joined to paclitaxel thus allowing selective delivery in vivo of the peptide-drug conjugate to those tumors in which EphA2 is overexpressed, and reaching improved paclitaxel efficiency [142,143,144]. A dimeric version of YSA peptide has also been developed and results able to activate receptor at low nanomolar concentrations, showing improved agonistic function with respect to monomeric YSA. When conjugated with paclitaxel, dimeric YSA shows higher efficacy at decreasing tumor metastasis [144]. Another group of potential EphA2-targeting compounds is represented by cyclic peptides. Phage display techniques coupled to medicinal chemistry efforts led to bicyclic peptides targeting EphA2. Among bicyclic peptides BCY6065 exhibits large affinity and selectively towards EphA2 LBD [145]. BCY6099 represents a chemically modified version of BCY6065 with enhanced physicochemical features, stability, and affinity that also displays strong anti-tumor action in in vivo tests [120,145].

Small molecules can be employed as alternatives to peptides and antibodies. Many small compounds targeting mostly EphA2 LBD and destroying the Eph-ephrin complex have been reported [97]. Among EphA2 antagonists there is lithocholic acid (LCA, (3a,5b)-3-hydroxycholan-24-oic acid), a secondary bile acid made through prokaryotic conversion of chenodeoxycholic acid [146]. Computational modelling studies have shown that LCA can insert inside the hydrophobic EphA2 LBD grove, thus mimicking ephrin A1 binding to EphA2 [147]. LCA indeed is able to compete reversibly with ephrin A1 for interaction with EphA2 but, it is unable to inhibit receptor kinase function [146]. However, LCA by targeting EphA2 LBD, is able to hamper receptor autophosphorylation induced by stimulation by ephrin A1-Fc in human prostate cancer cells and colon adenocarcinoma cell lines (PC3 and HT29) and avoids rounding and retraction of PC3 cells caused by EphA2 activation [97]. Starting from results on LCA, other compound inhibitors of the EphA2/ephrin complex have been designed and evaluated [97]. Amino acid residues have been chemically linked to LCA and have shown capacity to inhibit the interaction between EphA2 and ephrin A1 and to block phosphorylation of EphA2 in integral cells and reduce malignancy [147]. UniPR126 (N-(3a-hydroxy-5b-cholan-24-oyl)-L-tryptophan) is an LCA analogue with antagonistic properties, able to block EphA2 phosphorylation and angiogenesis in cultured cells at low micromolar concentrations [148]. UniPR126 has been recently employed as a cargo to generate a mixed nanomicellar delivery tool specific for EphA2 overexpressing tumor cells [149]. In particular, if loaded with the drug niclosamide, UniPR126-based mixed nanomicelles exhibit a substantial synergistic cytotoxic effect in PC3 cells [150]. In fact, in vivo studies in a PC3 xenograft model indicate 66.87% decrease of the tumor volume when nanomicelles loaded with niclosamide are employed with respect to niclosamide alone, which presents only halved activity [149]. UniPR129 is instead the L-homo-Trp conjugate of LCA and is capable of hampering EphA2-ephrin A1 binding [151]. Nevertheless, UniPR129 is able to hinder rounding of prostate cancer cells (PC3) and angiogenesis without exhibiting a cytotoxic action [97,151]. UniPR1331 has instead been generated by coupling L-tryptophan with a bioisostere derivative of LCA (i.e., 3β-hydroxy-D5-cholenic acid) [152]. UniPR1331 prevents EphA2 interaction with ephrin A1 with ten-fold enhanced potency with respect to the parent compound 3β-hydroxy-D5-cholenic acid. GBM growth can be blocked by administration of UniPR1331 that has also been shown to enlarge the progression time in a subcutaneous xenograft model by interfering with angiogenesis [97,152].

Many of the examples described above are related to EphA2 as kind of tumor-marker that can be exploited in drug delivery strategies by employing compounds targeting the LBD and eventually the receptor endocytosis mechanism. Another route to decrease tumor negative outcomes is related to compound inhibitors of receptor kinase activity [97]. Dasatinib for example is a TKI (Tyrosine Kinase Inhibitor) whose employment in myeloid leukemias is FDA approved [103]. Dasatinib presents sub-micromolar binding affinity for EphA2 and belongs to the class of compounds whose clinical trials are ongoing or approved (ponatinib and bosutinib are additional TKIs) [153]. Clinical trials have demonstrated dasatinib has a certain efficacy towards EphA2-correlated breast cancer and non-small cell lung cancer [154].

As an original alternative route to downregulate EphA2 levels and consequently its pro-oncogenic signaling, in our laboratory we are trying to attack an innovative target (i.e., the Sam domain of the receptor) through peptide/peptidomimetic inhibitors of its heterotypic Sam-Sam interactions with protein regulators of receptor endocytosis and stability.

## 4. EphA2-Sam and Its Interaction Network

EphA2 and all other Eph receptors possess a C-terminal Sam domain (Figure 5); EphA2-Sam has a canonical Sam fold characterized by a small five helix bundle (PDB entry 2E8N) (Figure 1a). Studies on Sam domains from diverse Eph receptors indicate that the Sam protein interaction module can induce diametrically opposed outcomes on receptor signaling.

EphA3 lateral interactions have been analyzed on the surface of live cells revealing that, in absence of ligand binding, EphA3 is able to form dimers and that the process is highly favored by contacts mediated by its Sam domain [155]. In particular, in an EphA3 deletion mutant lacking the Sam domain, ligand independent EphA3 dimerization results largely hampered. Moreover, tyrosine phosphorylation levels are lowered in EphA3 mutant where the Sam domain is deleted. It has been speculated that EphA3 activation process involves EphA3 unliganded dimers that play the role of intermediate forms. Following ligand stimulations these pre-formed dimers, along with EphA3 monomers, work as nucleation sites for the assembly of larger oligomers. Two EphA3 mutations related to lung cancer refer to EphA3 lacking partially or almost completely the Sam domain [156,157]. These mutants could favor pro-oncogenic signaling by hampering receptor dimerization and consequently decreasing tyrosine phosphorylated EphA3 levels in absence of ligand binding [155].

Anomalous EphA4 pathway has been linked to neurodegeneration but a correlation has also been established between EphA4 and tumor malignancy through a poorly clarified mechanism [158]. In melanoma large EphA4 mRNA expression has been related to poor patient survival and increased EphA4 signaling is thought to contribute to melanoma evolution. Mutation of Leucine 920 to Phenylalanine (L920F) in EphA4 Sam domain has been related to melanoma through enhanced EphA4 autophosphorylation. In detail, conformational variations in EphA4-Sam produced by L920F induce a change in EphA4 oligomeric forms letting receptor pass from dimeric to trimeric state; EphA4 trimers may assemble by exploiting unusual Sam domain interaction surfaces and upregulate receptor activation. Stimulation by ephrin ligand modulates differently EphA4 wild-type and the L920F mutant signaling. Thus, as concerning EphA4, enhanced activation due to the L920F mutation is linked to increased receptor oligomeric size and leads to enlarged pathogenesis [158].

In the case of EphA2, it has been shown that the Sam domain hampers receptor dimerization in absence of ligand and lowers EphA2 tyrosine phosphorylation contrarily to what observed for EphA3 [155,159]. HEK293 cells (that have low endogenous levels of EphA2) expressing an EphA2ΔS mutant, lacking the Sam domain, possess decreased cell motility with respect to cells expressing wild-type EphA2, due to improved dimerization of EphA2ΔS respect to the non-mutated receptor. In details, improved Y772 phosphorylation and decreased S897 phosphorylation have been observed in EphA2ΔS expressing cells [159]. Additional studies with the two deletion mutants: EphA2ΔKS (lacking both kinase and Sam domains) and EphA2ΔS, further point out the inhibitory role of EphA2-Sam towards receptor oligomerization [160]. Live cancer DU145 cells that stably express GFP (Green Fluorescence Protein)-labeled EphA2 mutants and tumor cells derived from mouse epithelia (i.e., 728 cells), where EphA1/EphA2 genes have been knocked out but with stable expression of GFP-labelled mutant receptors, have been employed to perform FCS (Fluorescence Correlation Spectroscopy) measurements. Enhanced oligomerization can be detected for EphA2ΔKS and EphA2ΔS respect to full-length EphA2 in both cell lines (DU145 and 728) [160] thus revealing that Sam domain inhibits unliganded dimers formation. Upon removal of Sam domain, receptor oligomerization triggers kinase activation inducing high tyrosine autophosphorylation levels. Experiments in 728 cell line following stimulation with ephrin A1-Fc show that interaction with ligand induces full length EphA2 oligomerization, although oligomers formed by the deletion mutants result much larger in size [160].

As concerning EphA4-Sam domain, differently from both EphA2 and EphA3, deletion of Sam domain is unable to induce significant outcomes on EphA4 signaling [161].

The above reported data, along with the observation that the ephrin ligand agonist YSA peptide (described in Section 3.1) is able to interfere with EphA2 pro-tumorigenic signaling by improving receptor dimers stability [162], let speculate that in the case of EphA2, the Sam domain may favor pro-oncogenic monomeric or low oligomeric forms downregulating receptor canonical kinase activation and support its targeting in anticancer drug discovery research. It has been speculated that interactions may exist in between the Sam and kinase domains of EphA2 that avoid dimerization or that binding between EphA2-Sam and other interaction partners could inhibit dimerization of the kinase domain [161]. The Sam domain of EphA2 is able to bind the Sam domain from the lipid phosphatase Ship2 (Ship2-Sam) [68,163,164] and the first Sam domain of the adaptor protein Odin (Odin-Sam1) [70,164]. In the phosphorylated form it is also able to interact with the SH2 domain of Grb7 (Growth factor receptor-bound protein 7) [165].

While the interaction in between EphA2-Sam and Ship2-Sam has been very well characterized from a structural and functional point of view [163,166], the functional meaning of the association between Odin-Sam1 and EphA2-Sam and the relevance of the interaction in the context of cancer cells need to be further clarified. Both Ship2 and Odin seem however to work by enhancing receptor upregulation either by inhibiting endocytosis [166] or ubiquitination and consequent degradation [167] as it will be better described below.

### 4.1. Ship2 (SH2-Containing 5′-Inositol Phosphatase)

Ship2 is a Phosphatidylinositol (PtdIn) 5-phosphatase which converts PI(3,4,5)P_3_ (i.e., PhosphatidylInositol(3,4,5)trisPhosphate) into PI(3,4)P_2_ (i.e., PhosphatidylInositol(3,4)bisPhosphate) by removing the phosphate group at the 5 position of the inositol unit [168,169]. PtdIns possess acyl chains through which they are engaged to the inner side of the plasma membrane [170]. A few PtdIns, like PI(4,5)P2, PI(3,4,5)P3 and PI(3,4)P2, play a pivotal role for triggering signaling cascades, as a variety of enzymes, including intracellular kinases and scaffolding proteins, can be selectively engaged by them for example through pleckstrin homology (PH) domains [170]. Ship2 plays first of all a well-known role in glucose metabolism [171], and it is considered a target for the treatment of type 2 diabetes, as, by downregulating PI(3,4,5)P3 levels, it suppresses PI3K-mediated insulin signaling pathway [172].

Nevertheless, Ship2 acts as modulator of the PI3K(PhosphoInositide 3-Kinase)/AKT signaling pathway whose relationship to cell survival, proliferation, effector function and vesicle trafficking is well established [170]. The proto-oncogene serine-threonine kinase AKT (also known as PKB) contains a PH domain able to interact with both PI(3,4,5)P3 and PI(3,4)P2 with different affinity: these two PtIns are necessary for full AKT activation [170]. The presence of both PI(3,4,5)P3 and PI(3,4)P2, through the enhanced AKT activation, supports cancer cells to escape inherent cell death machinery [170]. However, as concerning the correlation between Ship2 and cancer, it is controversial and dependent on the cancer cell type [168]. Ship2 levels are high in several breast cancer cell lines like MDA-231, where Ship2 over-expression favors cell survival, proliferation and tumor growth [171] while, Ship2 silencing causes anti-proliferative effects [168]. Generally, increased levels of Ship2 characterize glioma, breast and colon cancers and have been related to a poor prognosis due to improved cell migration and invadopodia maturation leading to enhanced metastatic capacity [170,171]. In contrast to these pro-cancer functions, Ship2 negatively regulates cancer cell progression in gastric cancer cell lines [171]. Similar to its role in gastric cancer, Ship2 also downregulates the growth of glioblastoma cells. Indeed, Ship2 overexpression is able to inhibit proliferation of U87-MG cell line, considered a model of glioblastoma, by producing a significant cell cycle arrest in the G1 phase [171].

The dual pro- and anti-oncogenic functions of Ship2 are likely related to the different levels of PI(3,4)P2 produced specifically by diverse cell types and by the large interaction network in which the protein is involved [168].

In fact, Ship2 includes within its modular domain arrangement, a part from the 5′-phosphatase catalytic domain, different protein interaction modules: Src homology 2 (SH2) domain, PH-R (Pleckstrin Homology Related) domain, C2 domain followed by a region containing binding motifs for SH3 and PhosphoTyrosine-Binding (PTB) domains (i.e., PXXP and NPXY, respectively) [168,169,170,171]. In addition, the C-terminal end of Ship2 includes an ubiquitin interacting motif (UIM) and a Sam domain which is also responsible for the heterotypic interaction with EphA2 [166].

#### 4.1.1. Ship2-Sam and Its Complex with EphA2-Sam: Structural and Functional Insights

In 2007 Zhuang and collaborators established a connection between the lipid phosphatase Ship2 and the EphA2 receptor. Ship2 is recruited at the EphA2 receptor site by means of a heterotypic Sam-Sam interaction to modulate ephrin-A1-induced EphA2 internalization and consequent degradation [166]. More in detail, Ship2 overexpression induces inhibition of receptor endocytosis following ephrin ligand stimulation while, Ship2 silencing causes receptor internalization and degradation. From a mechanistic point of view, it is speculated that EphA2 receptor is tyrosine-phosphorylated upon binding to its ephrin ligand and thus engages PI3K through the p85 subunit [166]. Recruitment of PI3K upregulates PI(3,4,5)P3 levels in turn leading to Rac1 GTPase activation that triggers endocytosis. Upon binding between EphA2 and ephrin ligand, Ship2 is also engaged by the receptor through Sam-Sam association, to downregulate PI(3,4,5)P3 levels and contrasting Rac1 activity and receptor endocytosis. The catalytic activity of Ship2 is involved into inhibition of endocytosis too as demonstrated by studies with a catalytically inactive Ship2 mutant [166]. Due to EphA2 overexpression in many types of cancers, as explained in Section 3, the process of ligand induced receptor endocytosis and the consequent degradation have been widely analyzed as potential routes to reduce tumor malignancy. The Ship2-Sam/EphA2-Sam interaction being linked to decreased endocytosis and upregulation of receptor levels should possibly produce pro-tumorigenic effects. In line with these observations, later studies with an EphA2-Sam mutant unable to associate with Ship2-Sam have shown that Ship2 works as inhibitor of the ligand induced EphA2 receptor activation while increasing the ligand independent promigratory function and thus it likely enhances the pro-oncogenic EphA2 route [163].

From a structural point of view, the interaction between EphA2-Sam and Ship2-Sam has been widely characterized by solution NMR techniques [68,163], X-Ray crystallography [164] and molecular dynamics simulations [173,174,175]. Analytical ultracentrifugation and ITC (Isothermal Titration Calorimetry) studies indicate formation of a dimer [68] while the value of the dissociation constant for the EphA2-Sam/Ship2-Sam complex resulted in the low micromolar range by diverse biophysical techniques [68,164,174] (K_D_ by ITC in PBS pH 7.7 = 0.75 ± 0.12 μM [68]; K_D_ = 2.2 ± 0.2 μM by SPR (Surface Plasmon Resonance) in Hepes pH = 7.4 [174]).

**Figure 6:**
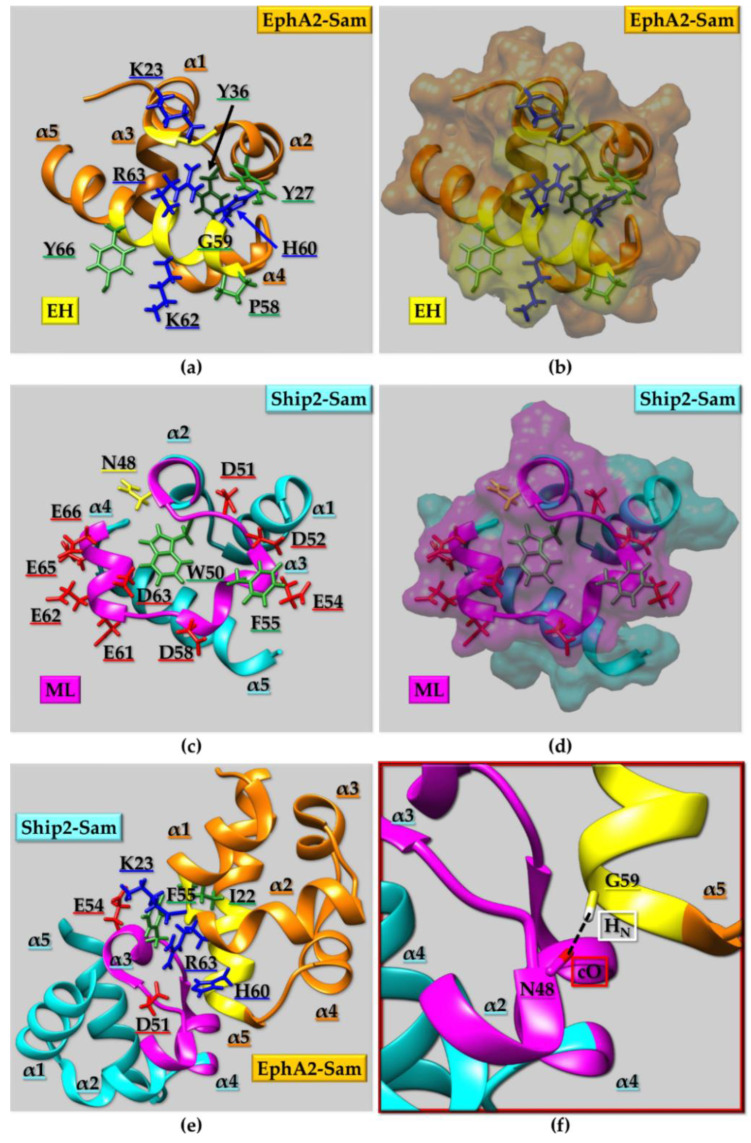
(**a**) Structure of EphA2-Sam (extracted from the EphA2-Sam/Ship2-Sam complex PDB entry 5ZRX [164], chain A). Ribbon representation of EphA2-Sam (orange) with the EH interface colored yellow. The side chains of positively charged residues present at the binding site for Ship2-Sam are shown in blue (i.e., K23, H60, K62 and R63, residues are numbered according to the PDB entry 2E8N referring to unbound EphA2-Sam, where K23 corresponds to K918 in the PDB entry 5ZRX). Green is used to highlight the aromatic and hydrophobic residues involved in complex formation (i.e., P58, Y66) along with additional tyrosine residues representing potential phosphorylation sites (i.e., Y27, Y36). (**b**) Surface representation of (**a**). (**c**) Structure of Ship2-Sam extracted from the EphA2-Sam/Ship2-Sam complex (PDB entry 5ZRX [164], chain A). Ship2-Sam is shown in the ribbon representation (cyan) and its ML region is highlighted in magenta. The side chains of negatively charged residues involved in complex formation (i.e., D51, D52, E54, D58, E61, E62, D63, E65 and E66 residue numbering is according to the PDB entry 2K4P [68] referring to free Ship2-Sam, where D51 corresponds to D1222 in the PDB entry 5ZRX), the polar residue N48 contributing as well to the interaction surface for EphA2-Sam is colored yellow, whereas aromatic residues (i.e., W50 and F55) are colored green. (**d**) Surface representation of (**c**). (**e**) Structure of the EphA2-Sam/Ship2-Sam complex (PDB entry 5ZRX [164], chain A). The side chains of a few residues providing crucial electrostatic and cation-π intermolecular contacts characterizing the ML/EH complex, are shown. (**f**) Zoomed view of the peculiar hydrogen bond that could anchor Ship2-Sam ML site to EphA2-Sam EH interface. The backbone carbonyl oxygen atom of N48 (Ship2-Sam) and the backbone amide proton of G59 (EphA2-Sam) are reported in red and white, respectively.

Both EphA2-Sam and Ship2-Sam possess the five-helix bundle fold characteristic of Sam domains and their association follows the ML/EH interaction model with EphA2-Sam providing the EH site while, Ship2-Sam forms the ML interface (Figure 6) [68]. In detail, the EH surface of EphA2-Sam (yellow in Figure 6a,b) covers most of the α5 helix (i.e., approximatively residues from P58 to Y66) and the α1α2 loop (i.e., residues from I22 to M24), whereas the ML surface of Ship2-Sam (highlighted in magenta in Figure 6c,d) encompasses mostly the amino-acid stretch from V46 to E66 thus including residues belonging to the C-terminal end of α2 helix, the α2α3 loop, the α3 helix, the α3α4 loop and the α4 helix [68,163,164]. As evident in Figure 6, EphA2-Sam EH site is positively charged and the ML site in Ship2-Sam is negatively charged thus, electrostatic interactions appear to play a major role in stabilizing the Sam-Sam association. For example, in the crystal structure of EphA2-Sam/Ship2-Sam complex, electrostatic interactions between K23 (EphA2-Sam) and E54 (Ship2-Sam) and, R63 (EphA2-Sam) and D51 (Ship2-Sam) can be clearly seen (Figure 6e) [164].

In addition, the presence of aromatic rings at the Sam-Sam interfaces favors the raise of cation-π contacts, like the one involving the R63 guanidinium group from EphA2-Sam and the F55 aromatic ring in Ship2-Sam. Interestingly, R63 guanidinium group in EphA2-Sam is also involved into a network of intermolecular H-bonds with H60 and D51 from Ship2-Sam, a part from forming an intramolecular H-bond with the backbone carbonyl oxygen of I22 (Figure 6e) [164]. In addition, the CH_2_ group of G59 in EphA2-Sam is positioned close to W50 in Ship2-Sam, and substitution of this residue with bulky amino acids leads EphA2-Sam to lose the ability to bind Ship2-Sam (Figure 6a,c, respectively) [163,164]. After adding hydrogen atoms to the crystal structure of the Ship2-Sam/EphA2-Sam complex, analyses of intermolecular contacts with programs like MOLMOL [176] and UCSF Chimera [177] suggest that the amide proton of G59 in EphA2-Sam is likely involved into hydrogen bonding with the carbonylic oxygen of N48 in Ship2-Sam, this kind of interaction, as already mentioned previously, can be considered a hallmark of Sam-Sam associations following the ML/EH topology (Figure 6f) [92].

Mutagenesis and structural studies highlight how the Ship2-Sam D51A/D52A double mutant lacks capacity to bind EphA2-Sam by affecting crucial electrostatic interactions with the EH region thus stressing out the key role of polar and electrostatic contacts in the formation of the EphA2-Sam/Ship2-Sam complex [163]. Further solution structural studies along with molecular dynamics simulations highlight the dynamisms of the Sam-Sam association characterized by fluctuations and possibility to assume diverse configurations [163,174,175].

Interestingly, EphA2-Sam contains 3 tyrosine residues (Y27, Y36, and Y66 in Figure 6a,b) representing phosphorylation sites [165]. CD (Circular Dichroism) and NMR studies show only a minor influence of phosphorylation on the structure and stability of EphA2-Sam; similarly, ITC analyses reveal small effect on the EphA2-Sam interaction affinity for Ship2-Sam [165]. This result is surprisingly since phosphorylation is a modification that introduces a negative charge in EphA2-Sam and thus should increase the repulsion between the receptor EH site and the negatively charged ML surface of Ship2-Sam. However, it is speculated that the dynamic nature of the EphA2-Sam/Ship2-Sam interaction surfaces, with ability to assume different configurations, can somehow counterbalance the charge repulsion and lower its effect on the binding affinity [165].

Once clarified structural features characterizing Ship2-Sam/EphA2-Sam association, mutant EphA2-Sam forms with different abilities to interact with Ship2-Sam were employed to clarify the role of EphA2-Sam within receptor related pathways. In detail, R56T EphA2-Sam, with improved binding affinity for Ship2-Sam, and K23E/P58A/K62E EphA2-Sam, unable to consistently interact with Ship2-Sam, were assayed in a cellular context [163]. In HEK293 cells, a significant increase in ligand-induced EphA2 activation with enhanced receptor degradation rate was detected for the K23E/P58A/K62E mutant, which possessed a decreased ability to bind Ship2-Sam with respect to wild-type EphA2. On the contrary, a moderate increase in the EphA2 cellular levels was observed for the R56T mutant, further indicating that the stronger binding to Ship2-Sam favors inhibition of receptor endocytosis and consequent degradation [163]. Surprisingly, despite the two mutants have opposite capacity to interact with Ship2-Sam and influence in different ways ligand induced EphA2 endocytosis and degradation, they both, when over-expressed in cell, exert a similar reduction of ligand-independent cell migration with respect to wild-type EphA2 [163]. These data indicate that regulation of the ligand-independent cell migration pathway by Ship2-Sam engagement to EphA2-Sam can be reached only by a fine tuning of the interaction strength and thus by avoiding too strong or too weak Sam-Sam interaction affinities.

### 4.2. The Adaptor Protein Odin

Odin (also named Anks1a) belongs to the ANKS protein family and includes within its modular domain organization six ankyrin repeats located at the N-terminus, two tandem Sam domains (i.e., Sam1 and Sam2) in the middle region, and a PTB domain positioned at the C-terminus [178,179].

Odin interacts with many proteins involved in diverse cellular processes, including molecules playing pivotal roles in modulating receptor endocytosis [180]. Odin is considered an adaptor signaling protein which works downstream of different RTKs (e.g., EGF and Eph receptors) [178,181]. For example, EGFR routing to the lysosome and EGF-induced EGFR internalization from the plasma membrane into recycling endosomes are two processes inhibited and enhanced by Odin, respectively [181].

Odin has also been described as a substrate for Lymphocyte cell-specific protein-tyrosine kinase (Lck), a member of the Src family kinases that is aberrantly expressed in a few colorectal cancers [179]. Nevertheless, a role in ErbB2 (Erythroblastic oncogene B2) signaling has been attributed to Odin to support the exit of the EphA2/ErbB2 complex from the ER (Endoplasmic Reticulum). EphA2 binding to ErbB2 amplifies ErbB2 signaling and promotes breast tumorigenesis. Odin exploits its ankyrin repeats region to interact with the kinase domain of EphA2 and favor receptor accumulation at ER exit sites [178].

Odin has been first linked to EphA8 mediated signaling pathway through an interaction between its PTB domain and the EphA8 juxtamembrane domain [182]. Interestingly, Odin through its Sam domains enhances the stability of EphA2 and EphA8 receptors as will be better described below [167].

#### 4.2.1. Odin-Sam1 and Its Complex with EphA2-Sam: Structural and Functional Insights

In 2010, Kim and co-workers established a connection between Sam domains of Odin and EphA receptors signaling [167]. In vitro studies in HEK293T cells showed that Sam domains of Odin played a pivotal role in hampering degradation of EphA8 following ligand stimulation. Nevertheless, upon Odin silencing, the decrease of cell migration induced by EphA8 ligand stimulation appeared highly attenuated. Stability of EphA8 resulted to be modulated by Odin Sam domains as, over-expression of an Odin mutant form lacking Sam domains induced a fast decay of EphA8 levels following ligand treatment. In addition, Odin upregulation induced decrease of EphA8 ubiquitination, occurring upon ligand stimulation, whereas the over-expression of the Odin mutant deprived of Sam domains increased ligand-mediated ubiquitination of EphA8. From a mechanistic point of view, it is speculated that ubiquitination of EphA8 receptor on the cell surface by the Cbl E3 ligase occurs following ephrin ligand binding, leading to fast receptor degradation. Odin Sam domains however exhibit a stronger binding to the ubiquitinated receptor with respect to non-ubiquitinated EphA8, thus blocking additional ubiquitination with consequent receptor stabilization. Otherwise, it is also possible that Odin might improve EphA8 stability through different routes like modulation of deubiquitination pathways [167].

Additional in vitro cell-based assays demonstrated a similar regulatory role of Odin towards EphA2. In fact, upon Odin silencing in MDA-MB-231 breast cancer cells a rapid decrease of EphA2 levels following ligand stimulation could be revealed thus confirming the protective role of Odin towards EphA2 degradation in cells. In addition, while ephrin-A5 treatment of MDA-MB-231 cells induced reduction of cell migration through EphA2 ligand-dependent route, Odin silencing was able to reduce this outcome. Cell-based assays with an Odin mutant form lacking Sam domains clearly pointed out that Odin regulated cell migration and stability of EphA2 through its Sam domains as also evidenced for EphA8. Finally, studies in Odin-deficient mice further confirmed that Odin absence led to decreased inhibition of cell migration induced by ephrin-A5 stimulation [167].

This study, showing the important role of Odin Sam domains in enhancing EphA2 stability [167], quickly attracted our attention because of its similarity with the Ship2-Sam domain, which promotes receptor stability by inhibiting endocytosis [166].

**Figure 7:**
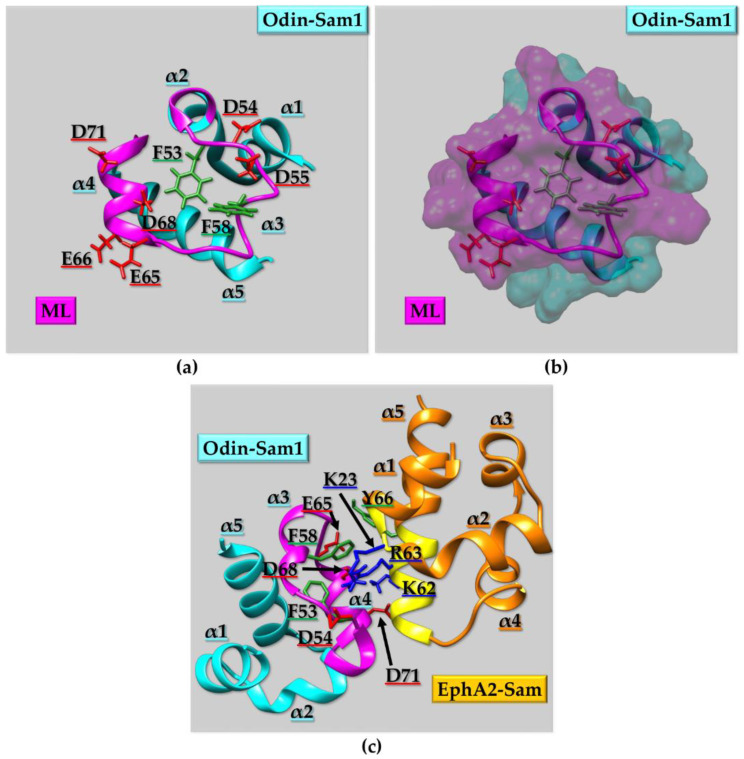
(**a**) NMR structure of Odin-Sam1 (PDB entry 2LMR [70], conformer n.1). The flexible N- and C-terminal tails (M21-Q30 and P91-N101) are not shown. Odin-Sam1 is reported in the ribbon representation (cyan) with the ML region highlighted in magenta. The side chains of negatively charged (D54, D55, E65, E66, D68 and D71) and aromatic residues (F53 and F58) present in the ML surface are reported in red and green, respectively. (**b**) Surface representation of (**a**). (**c**) One representative docking pose of the Odin-Sam1/EphA2-Sam complex [70]. The side chains of residues providing crucial intermolecular contacts characterizing the ML/EH complex are shown in a neon representation with heavy atoms and polar hydrogens.

Comparative analyses of primary sequences indicated rather high sequence homology between Ship2-Sam and the first Sam domain of Odin (Odin-Sam1) [70]. NMR studies conducted in our laboratory revealed for Odin-Sam1 a canonical Sam fold (Figure 7a,b) and a low tendency to self-associate in solution in analogy to what described for Ship2-Sam and EphA2-Sam [68]. Moreover, interaction studies by NMR, SPR and ITC indicated the ability of Odin-Sam1 to bind EphA2-Sam by forming a dimer (dissociation constant K_D_ = 5.5 ± 0.9 μM by SPR and K_D_ = 0.62 ± 0.04 μM by ITC). NMR Chemical Shifts Perturbation (CSP) experiments pointed out formation of a ML/EH complex. Odin-Sam1 provides in this case the negatively charged ML site and competes with Ship2-Sam for a common bind site on the surface of EphA2-Sam, which instead forms the positively charged EH site [70].

A model of EphA2-Sam/Odin-Sam1 complex was built based on NMR CSP results with the Haddock webserver and showed a very similar structural topology with respect to the one observed for the Ship2-Sam/EphA2-Sam association (Figure 7c) [70]. EphA2-Sam EH binding region includes approximatively residues from P58 to Y66 (α5 helix) and residues from I22 to M24 (α1α2 loop). Odin-Sam1 central region, encompassing mainly α3, α4 helices, α2α3 and α3α4 loops, forms the ML site (residue range ~ from L49 to G73) (Figure 7c). Intermolecular contacts at the Sam-Sam interfaces are provided by K23, K62, R63, Y66 from EphA2-Sam EH site and F53, D54, F58, E65, E66, D68, D71 from the ML region of Odin-Sam1 (Figure 7c). The complex is stabilized mainly by H-bonds and salt-bridges in between positively charged residues of EphA2-Sam and negatively charged residues of Odin-Sam1 along with cation-π and π-π contacts (Figure 7c) [70].

Structural studies demonstrated that the two Sam domains of another ANKS family protein belonging to the same Odin family, named AIDA-1b (also known as Anks1b), bind to each other forming a ML/EH complex where AIDA-1-Sam1 and -Sam2 form the ML and EH interfaces, respectively [89]. The high sequence homology between tandem Sam domains from AIDA-1 and Odin [70] let speculate that Odin-Sam1 and -Sam2 may similarly interact with each other forming a ML/EH complex. In this scenario, uncoupling of the tandem Sam domains of Odin should be necessary to allow binding of Odin-Sam1 to EphA2-Sam but more studies are necessary to prove this hypothesis along with the relevance of the diverse Sam-Sam interactions within a cellular context [70].

Further studies demonstrated the ability of Odin-Sam1 to bind in addition to EphA2-Sam, also EphA1-Sam and EphA6-Sam [164]. The crystal structure of the Odin-Sam1/EphA6-Sam complex [164] is characterized by a ML/EH structural arrangement that appears very similar to that characterizing the Odin-Sam1/EphA2-Sam complex, with Odin-Sam1 contributing the ML site while EphA6-Sam like EphA2-Sam forming the EH interface.

## 5. Peptides Targeting EphA2-Sam and Its Interactome

Peptides and peptidomimetics are considered advantageous tools to modulate protein-protein networks involved in diseases, as they could chemically and structurally mimic interaction surfaces and target protein pockets and grooves better than small molecules [183]. However, Sam domains mediated associations, as most protein-protein interactions (PPIs), are characterized by large and flat binding regions and consequently, they are very challenging to target even with peptides. Actually, to date, at the best of our knowledge, only a few studies, centered on peptides targeting Sam domains, have been described in literature. An example is provided by the work by Neira and collaborators that is focused on peptides from the Sam domain of the p73 protein [184,185]. Another work describes instead peptides against the Sam domain of DLC1 that are able to modulate its interactome [186].

In the previous paragraphs of this review, we described the Sam domain of EphA2 receptor from a structural and functional perspective and reported on its interactions with Sam domains from the proteins Ship2 and Odin, which are potentially relevant in cancer cells pathways. Considering that inhibition of heterotypic Sam-Sam interactions involving EphA2 could be a promising route in cancer therapy, during the last few years, we centered our research activities on the design of peptides able to hamper these interactions. To achieve our goal and obtain such peptides we implemented a variety of approaches: (1) we set-up protein dissection strategies involving all Sam domains under study (i.e., EphA2-Sam, Ship2-Sam and Odin-Sam1) [187,188,189,190], (2) we designed helical peptides, both linear and stapled, to mimic native-like structural motives in Sam domains [191,192], (3) we implemented structure-based computational approaches to generate virtual peptide libraries to be screened in silico against target Sam domains [92,193]. All designed peptides were evaluated through multidisciplinary studies involving diverse computational and experimental techniques such as NMR and CD (Circular Dichroism) for conformational studies, molecular modelling (docking), NMR, ITC, SPR and MST (Microscale Thermophoresis) for binding analyses, and in vitro cell-based assays for evaluation of biological activities.

The different implemented strategies (Table 2) and identified Sam ligands (Table 3) will be deeply described below.

### 5.1. Protein Dissection Approaches

The first strategy we adopted to discover novel ligands inhibiting formation of the EphA2-Sam/Ship2-Sam and EphA2-Sam/Odin-Sam1 complexes was the protein dissection approach. This approach is focused on the design and evaluation of peptide sequences reproducing ML and EH binding interfaces, thus including the hot spot residues mainly responsible for Sam-Sam association.

#### 5.1.1. Mid-Loop Peptides

**Figure 8:**
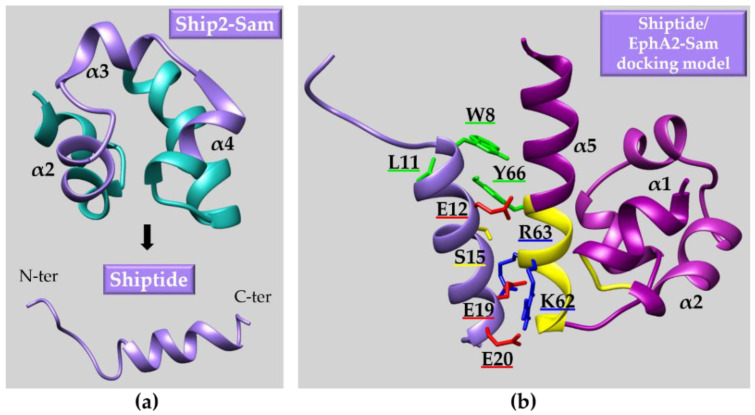
Ship2-Sam protein dissection approach. (**a**) NMR structures (conformers n.1) of Ship2-Sam (PDB entry 2K4P [68]), with the region encompassed by the Shiptide colored violet (top), and Shiptide calculated in 70% TFE (bottom). (**b**) The best docking model (i.e., the one with the lowest score) of the Shiptide (violet)/EphA2-Sam (magenta) complex is reported with the EphA2 EH region highlighted in yellow [187]. Docking studies were conducted with Haddock [196,197]. The side chains of a few residues performing intermolecular contacts are shown (positively charged, negatively charged, aromatic and polar amino acids are colored blue, red, green and yellow, respectively). Residues involved into H-bonding are K62, R63 from EphA2-Sam α5 helix and E19, E20 from Shiptide. Non-bonded intermolecular contacts are provided by residues P58, G59, K62, R63, Y66, G70 from EphA2-Sam α5 helix and W8, L11, E12, S15, E19, E20, L22 from the Shiptide.

As already mentioned in Section 4.1.1 EphA2-Sam interacts with Ship2-Sam following the ML/EH structural topology in which the ML interface is represented by the central regions of Ship2-Sam (Figure 6c–e). The Shiptide is a 22 residue long peptide whose sequence (Table 2) encompasses residues from E43 to L64 of Ship2-Sam (residue numbering according to PDB entry 2K4P [68]) thus comprising most of its ML site (Figure 8a) [68,69,187]. First, to gain insights into the Shiptide tendency to adopt a native-like fold, when extracted from the whole 3D protein organization, CD and NMR studies were conducted in 10 mM sodium phosphate buffer at pH 7.2. Not surprisingly, the Shiptide resulted disordered in an aqueous environment. Next, as it is very challenging to collect transfer NOE data and determine a bound peptide conformation while working with small proteins like Sam-domains [194], the co-solvent 2-2-2-trifluoroethanol (TFE) was employed to investigate the ability of the peptide to assume precise secondary structure elements that could mimic bioactive conformations [195]. A solution structure of the Shiptide was calculated in a H_2_O/TFE mixture containing 70% TFE because, as indicated by CD studies, that amount of co-solvent should allow the peptide to assume the highest structuration level [187]. The Shiptide NMR structure in TFE is characterized by an α-helix encompassing most of peptide sequence except for the more disordered stretch at the N-terminus (Figure 8a). The ordered portion of the peptide comprises the amino acid sequences corresponding to the α3 and α4 helices of the intact Sam domain, but it is also extended to the short unstructured interhelical loops (Figure 8a) [187]. Interaction studies by different techniques such as NMR, ITC and SPR let speculate a weak binding and a dissociation constant in the micromolar range for the complex with EphA2-Sam [187,193]. The Shiptide/EphA2-Sam association was next analysed in silico by molecular docking techniques employing the NMR structures of Shiptide, calculated in TFE, and of EphA2-Sam (PDB entry 2E8N) (Figure 8b) [187]. Analyses of docking poses indicated that the Shiptide helical arrangement has a certain tendency to face the EphA2-Sam α5 C-terminal helix, which represents a crucial element of the EH interacting area for Ship2-Sam [187], resembling the tail-to-tail Sam/Sam interaction observed in the crystal structure of oligomeric EphB2-Sam (PDB entry 1B4F [84]). The most representative model of the EphA2-Sam/Shiptide complex (i.e., the one with the lowest(= best) docking score) is an example of this tail-to-tail topology of binding (Figure 8b), and is stabilized by electrostatic contacts between positively charged residues from EphA2-Sam (K62 and R63) and negatively charged residues from Shiptide (E19 and E20—corresponding to E61 and E62 in the Ship2-Sam PDB entry 2K4P [68]), together with several non-bonded contacts, many of which occurring between EphA2-Sam Y66 and the side chains of different Shiptide residues (Figure 8b). Based on this computational study the design of Shiptide analogues with improved binding affinity to EphA2-Sam can be envisioned.

**Figure 9:**
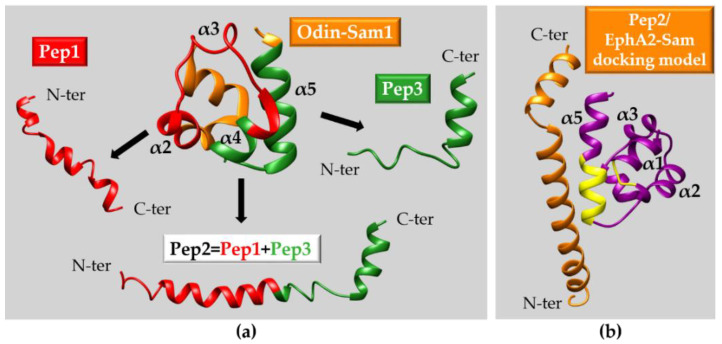
(**a**) Odin-Sam1 protein dissection strategy. The Odin-Sam1 NMR structure (PDB entry 2LMR [70], conformer n.1) is reported in orange with the regions included in Pep1 and Pep3 colored red and green, respectively. Pep2 derives by combination of Pep1 and Pep3 fragments. Representative NMR conformers (i.e., n.1 of each NMR ensemble) of Pep1, Pep2 (PDB entry 2MYQ [188]) and Pep3, calculated in H_2_O/TFE mixtures, are also shown. (**b**) Best docking model of the Pep2 (orange)/EphA2-Sam (violet) complex with the EphA2 EH region highlighted in yellow. Docking calculations were carried out with Haddock [196,197] and the NMR structures (first conformers) of Pep2 (PDB entry 2MYQ) and EphA2-Sam (PDB entry 2E8N). EphA2-Sam EH residues involved in interaction with Odin-Sam1 having high solvent accessibility (K23, R56, G59, H60, K62, R63, Y66) were set as active, and N-/C-terminal disordered protein regions were considered as fully flexible [70]. All Pep2 residues present solvent accessibility ≥30% and were set as active. The program MOLMOL [176] was used to assess solvent accessibility for both protein and peptide. The docking protocol calculated 1000 structures with a rigid body energy minimization in a first step then, the best 200 solutions were subjected to a semi-flexible simulated annealing and a final refinement in water. Best solutions underwent a clusterization protocol with a RMSD cut-off equal to 5 Å [196,197].

As discussed in Section 4.2.1 Odin-Sam1 also binds EphA2-Sam by adopting a ML/EH model similar to that observed for the interaction between EphA2-Sam/Ship2-Sam (Figure 7) [70] thus, a protein dissection approach relying on Odin-Sam1 ML interface was also investigated. Three different peptides were designed: Pep1, Pep2 and Pep3 (See Table 2 and Figure 9a) [188]. Pep1 primary sequence encompasses most of the ML interaction region of Odin-Sam1 for EphA2-Sam; Pep2 comprises the whole Pep1 fragment but, it is C-terminally elongated to include also the Odin-Sam1 sequence corresponding to the α4α5 loop and the α5 helix; these additional peptide portions, although external to the binding site for EphA2-Sam, were added to eventually improve peptide structuration and induce a more native-like fold through establishment of additional inter-residue contacts (Figure 9a). Finally, Pep3 represents a control peptide that reproduces only the portion of Pep2 external to the Odin-Sam1 ML interface [188] (Figure 9a). Conformational studies were carried out by CD and NMR techniques in sodium phosphate buffer and TFE/H_2_O mixtures. Based on the results of CD studies performed with increasing amounts of TFE, NMR structure characterization was carried out in presence of 70% TFE for Pep1 and Pep2 and 40% TFE for Pep3 as, this last peptide exhibited a larger tendency to assume a helical conformation even in presence of low TFE amounts. In line with results collected for the Shiptide, Pep1 NMR structure is characterized by a single rather irregular helical segment, where the helical content spans regions that are helical in the intact Odin-Sam1 domain (i.e., residues deriving from α2, α3, and α4 helices) but also extends to the interhelical segments that are unstructured in the whole Sam domain (Figure 9a). Instead, the Pep2 NMR structure is composed of two almost orthogonal α-helical segments separated by bend-turn motives; the first helix covers the region encompassing the Odin-Sam1 ML interface where native and non-native helical segments are present, whereas the second one is formed by the segment corresponding to the α5 helix of Odin-Sam1, which assumes in the peptide a native-like structuration (Figure 9a). Pep3 NMR structure is characterized by a disordered N-terminal stretch followed by a native-like helical conformation encompassing the sequence corresponding to the Odin-Sam1 α5 helix (Figure 9a). Following conformational analyses, interaction studies were conducted by SPR techniques revealing the capacity of Pep2 to bind EphA2-Sam with a dissociation constant in the high micromolar range (Table 3) [188]. Thus, to obtain further insights on the interaction mode of Pep2 and EphA2-Sam, docking studies were performed with the Haddock webserver [198]. Very different binding poses could be obtained (only 33,5% of docking solutions were grouped in 8 clusters of related structural poses) and visual inspection of docking results pointed out that the two-helices structural arrangement of Pep2 is unable to well target the EH site. For example, in the complex with the best docking score, only the second Pep2 C-terminal helix, along with the interhelical bend and the C-terminal portion of the first helix provide a certain number of intermolecular interactions with EphA2-Sam residues, which are included both inside and outside the EH site, while the more N-terminal Pep2 region remains largely exposed (Figure 9b).

Interestingly, although Pep3 comprises an Odin-Sam1 region located outside the EH interaction site for EphA2-Sam, SPR studies indicated some very weak residual binding to the receptor (Table 3). We speculated that this outcome could be related to the residual helical conformation characterizing this peptide that could provide a tail-to-tail like interaction with Sam domains [188]. To further address this issue, as will be better described in Section 5.3.2, Pep3 NMR structure was employed in molecular modelling studies to point out residues potentially important for binding to EphA2-Sam and design a hydrocarbon stapled helical Pep3 analogue [192].

Noticeably, the Ship2-Sam and Odin-Sam1 dissection approaches indicate that peptides encompassing ML regions are weak binders of EphA2-Sam EH interface, probably due to their disordered nature and the inability to adopt in aqueous environment native-like conformations when extracted from the parental protein.

#### 5.1.2. End-Helix Peptides

**Figure 10:**
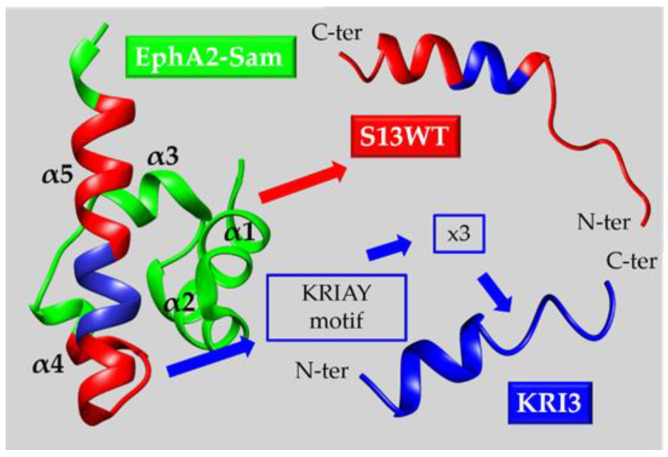
EphA2-Sam protein dissection approach. The first conformer of the EphA2-Sam NMR structure (PDB entry 2E8N) is shown with the region enclosed in the S13WT peptide colored red and the “KRIAY” motif evidenced in blue. This motif repeated three times in tandem corresponds to the sequence of the KRI3 peptide. The NMR structures (conformers n.1) of S13WT (PDB entry 5NZ9) and KRI3 calculated in presence of 60% and 50% TFE [189], respectively are also reported.

EphA2-Sam with its EH interface (Figure 6a,b) was the focus of another protein dissection approach [189]. In this case several peptides encompassing the EH site and adjacent regions were designed (Table 2). S13WT reproduces the EphA2-Sam α5 C-terminal helix along with a portion of α4 and the α4α5 loop (Figure 10) (Table 2). The S13-SS peptide was designed to better mimic the discontinuous epitope of the EH region and is composed by two peptide fragments joined together by a disulfide bridge (Table 2). In S13-SS, the first fragment encompasses the identical S13WT sequence, whereas the second one includes the α1α2 loop region of EphA2-Sam, which in the intact domain provides additional intermolecular contacts with Ship2-Sam and Odin-Sam1 ML sites (Table 2) [189]. In addition, we noticed on the α5 helix of EphA2-Sam the “KRIAY” motif including positively charged and aromatic residues. As both negatively charged and aromatic residues important for the binding to EphA2-Sam are present on the ML interface of Ship2-Sam and Odin-Sam1 (Figure 6c,d and Figure 7a,b), the “KRIAY” motif could represent a “hot-spot” stretch determining complex formation and was employed to design further peptides. The short peptide KRI contains the “KRIAY” motif alone while, KRI2 and KRI3 peptides include the same motif repeated twice and thrice in tandem (Table 2), respectively, to eventually favor additional intermolecular contacts.

Conformational analyses of all peptides were conducted by CD and NMR spectroscopies in aqueous environment, where all peptides resulted mainly disordered, and in presence of increasing concentrations of the co-solvent TFE. CD titration experiments revealed again the amount of TFE needed to reach in the peptides the highest structuration level. NMR studies showed for S13WT peptide a native like helical conformation in presence of 60% TFE (Figure 10) [189]. Docking studies were later conducted with the S13WT NMR structure to gain information useful for the design of a stapled peptide (S13ST) (See Section 5.3.2) [192].

The KRI3 peptide in presence of 50% TFE assumes, as shown by NMR structure calculations, a helical conformation extending through the whole peptide sequence (Figure 10) [189]. This KRI3 NMR structure was employed in docking studies thus collecting structural data for the design of KRI3 peptide analogues (See next Section 5.2).

Interaction assays were performed by NMR, SPR and MST and clearly revealed that KRI3 was the best Ship2-Sam ligand among this peptide series modelled on EphA2-Sam EH site (Table 3). Detailed NMR experiments indicated that KRI3 mainly targets the ML region and when employed at large concentrations, is able to compete with EphA2-Sam for a common binding site on the surface of Ship2-Sam. The KRI3 peptide was also tested in cell viability assays and it showed to be more cytotoxic to PC3 prostate cancer cells than to normal human dermal fibroblasts [189].

### 5.2. KRI3 Analogues

**Figure 11:**
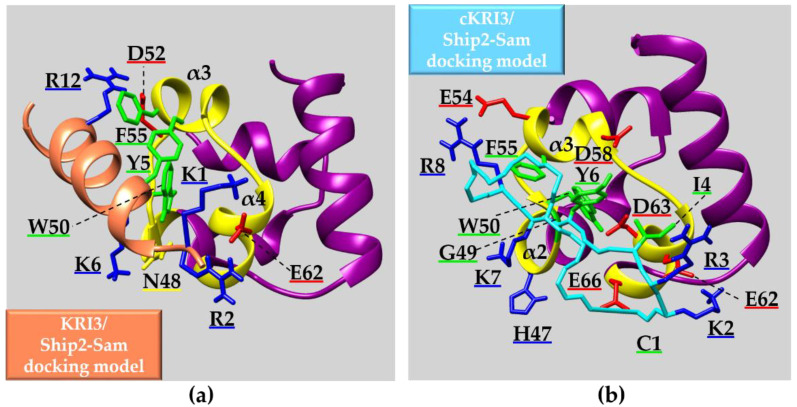
(**a**) Best ranked docking pose of the KRI3/Ship2-Sam complex [190] with the Ship2-Sam ML region highlighted in yellow. Side chains of residues providing intermolecular contacts are shown. N48 (α2 helix), D52 (α2α3 loop), F55 (α3 helix), E62 (α4 helix) of Ship2-Sam along with K1, R2, Y5, K6, R12 of KRI3 participate in H-bonding. The side chains of Ship2-Sam F55 and W50 make non-bonded contacts with Y5 of KRI3. (**b**) Best docking solution of the cKRI3/Ship2-Sam complex. The ML interface is colored yellow and residues involved in intermolecular H-bonding are indicated: H47, G49, E54, D58 (α3 helix), E62, D63, E66 (α4 helix) of Ship2-Sam and C1, K2, R3, I4, Y6, K7, R8 of cKRI3. Protein residues W50 and F55 perform non-bonded interactions with Y6 of cKRI3. Docking studies were conducted with the Haddock webserver [196,197]. The residues of Ship2-Sam set as active during docking calculations were chosen based on results from NMR binding studies, among those with solvent exposure ≥30% undergoing major chemical shift and/or intensity changes upon interaction with the peptide, and belonging to the ML region (H47, L53, E54, E61, E66, A67) [190]. As concerning the peptide, all residues were provided with high solvent exposure (equal to at least 30% as calculated with MOLMOL [176]) and were set as active. In both (**a**,**b**) panels side chains are colored blue, red, green and yellow for positively charged, negatively charged, hydrophobic and polar amino acids, respectively, only heavy atoms and polar hydrogens are displayed.

The protein dissection approach focused on EphA2-Sam led to the identification of the KRI3 peptide as a ligand of Ship2-Sam (Section 5.1.2, Table 2 and Table 3). Based on results from NMR chemical shift perturbation experiments highlighting the Ship2-Sam residues mainly involved in interaction with KRI3, and having in hand NMR structures of both protein and peptide, we performed docking studies (Figure 11a) [189,190]. Analyses of different docking poses further confirmed the importance of KRI3 Lysine, Arginine and Tyrosine residues in providing stabilizing intermolecular interactions with the negatively charged and aromatic residues present on the Ship2-Sam ML surface. Indeed, in the best scoring docking solution interactions occur between KRI3 positively charged residues (K1, R2, K6, R12) and Ship2-Sam negatively charged residues (D52, E62), while Y5 (KRI3) is involved in intermolecular contacts with W50 and F55 (Ship2-Sam) (Figure 11a). Results from docking studies supported the design of linear and cyclic KRI3 analogues [190]. In detail, considering the importance of Tyrosines for the protein/peptide interactions in docking models, the KRI3-YM peptide in which Tyrosines were mutated into Alanines was generated as negative control (Table 2). In an attempt to increase the number of electrostatic interactions in the KRI-IM peptide, the Isoleucines, which according to docking solutions appeared less involved in interactions with Ship2-Sam, were mutated in Lysines (Table 2). To evaluate if the addition of an extra “KRIAY” motif to the KRI3 sequence could enhance binding affinity to Ship2-Sam, the KRI4 peptide was designed (Table 2). Finally, to modulate peptide flexibility cyclic analogues of KRI3 (cKRI3) were obtained by adding Cysteine residues at N- and C-termini of KRI3 sequence and achieving cyclization through a disulfide bridge. Cyclic peptides were conceived with and without terminal protections to further evaluate modulation of the binding to Ship2-Sam by N- and C-terminal peptide charges (Table 2) [190].

Interaction assays between different KRI3 analogues and Ship2-Sam were conducted by different techniques (NMR, SPR and MST). As expected, no binding was revealed between KRI3-YM and Ship2-Sam. As concerning KRI4, NMR titration experiments clearly pointed out unspecific interactions with Ship2-Sam as reduction of protein signal in NMR spectra and appearance of a precipitate in the NMR sample was soon evident even at the lowest peptide concentrations. This effect, which was revealed at much lower extent for KRI3, is possibly due to the increased positive charge of the peptide inducing saturation of negative charges positioned around the protein surface in an unspecific manner, thus decreasing the solubility of the protein/peptide complex (Table 2) [189,190].

In spite of the increased positive charge, KRI3-IM peptide is unable to bind Ship2-Sam better than KRI3 (Table 3) and moreover, it presents a reduced stability in FBS (Fetal Bovine Serum) [190].

Similarly, cyclization does not improve binding affinity toward Ship2-Sam with respect to linear KRI3. Indeed for a few of the cyclic KRI3 analogues, for instance Ac-cKRI3 provided with N-terminal Acetyl protection, severe unspecific interactions, also possibly linked to peptide aggregation, were detected. In fact, it was possible to obtain a quantitative estimate of the binding to Ship2-Sam only for cKRI3 (lacking N- and C-terminal protections) that resulted comparable to what observed for KRI3/Ship2-Sam interaction (Table 3). Cyclization improves the serum stability of cKRI3 with respect to linear counterpart although preliminary cellular uptake experiments in PC3 cells demonstrate formation of peptide aggregates hampering cell entry at difference from the KRI3 linear peptide for which cell entry can be clearly detected [190].

Conformational analyses of all KRI3 analogues were performed as well. CD studies revealed that the peptides are all disordered in aqueous environment. KRI3-IM has a tendency to remain unstructured even in presence of high TFE amounts. The NMR solution structure of cKRI3 peptide was calculated in a mixture of H_2_O/TFE (50/50 *v*/*v*). cKRI3 solution structure is characterized by a flexible cyclic arrangement, and it can be better represented by different clusters of conformational related families [190]. Docking studies were conducted by employing the NMR structures of cKRI3 (i.e., the representative conformer of the most populated cluster [190]) and Ship2-Sam (Figure 11b). The cKRI3/Ship2-Sam complex, similarly to KRI3/Ship2-Sam association, is stabilized by several H-bonds involving cKRI3 positively charged residues (K2, R3, K7, R8) and Ship2-Sam negatively charged residues located on the ML interface (E54, D58, E62, D63, E66); in contrast, Y6 in cKRI3 and W50 and F55 in Ship2-Sam are involved into non-bonded interactions (Figure 11b).

Taken together results collected thus far on KRI3 analogues provide useful information for the design of additional and possibly more effective cyclic and linear peptide ligands targeting Ship2-Sam ML site and suggest routes to enhance peptide stability for cell-based analyses.

### 5.3. Helical Peptides

Helical peptides are a prominent class of anti-cancer molecules, and share different characteristics with antimicrobial peptides, concerning either the secondary structure and the mechanisms adopted to induce cells death. Indeed, anti-cancer helical peptides are generally rich in positively charged amino acids and possess an amphipathic helical structure that allows them to penetrate plasma and nuclear membranes and induce cancer cell apoptosis by different mechanisms such as interference with DNA synthesis and cells division, or cell disruption through membrane lysis and consequent apoptotic cell death via caspase-3 dependent action [199]. Since cancer cell membranes, differently from normal cells, expose negatively charged components, the positive net charge of anti-cancer helical peptides makes them selective towards pathological cells [199]. Moreover, considering that helices are structural motives frequently found at protein-protein interfaces, helical peptides, by mimicking native motives, could act as proper ligands of target proteins. A strategy to force a peptide to assume a helical conformation is the stapling approach, which relies on the introduction into the primary sequence of non-natural amino acids whose side chains are covalently linked. Residues involved in the stapling are generally located in positions i, i+4 or i, i+7 so that the bridge they form is of the right length to induce a helical turn [34]. This approach is useful to overcome a few issues related to the use of peptides as drugs. In fact, the presence of the stapling decreases peptides susceptibility to proteases attack and allows them to better penetrate cell membranes as the helical structural arrangement generally leads to exposure of non-polar residues thus enhancing peptide hydrophobicity [34]. Therefore, stapled peptides are optimal candidates to inhibit protein-protein interactions linked to pathological conditions such as cancer [200,201,202,203].

As protein dissection approaches demonstrated that linear disordered peptides represent weak blockers of Sam-Sam interactions involving EphA2-Sam, helical peptides targeting Sam domains were designed by two diverse strategies as described below.

#### 5.3.1. Helical Linear Peptides

Sam-Sam interactions mediated by EphA2-Sam are largely driven by electrostatic contacts involving a positively charged spot in EphA2-Sam (i.e., EH site) and the negatively charged surfaces (i.e., ML sites) in Ship2-Sam and Odin-Sam1. The C-terminal α5-helix is an important interaction element in Sam domains, indeed it is included in EH sites, like in the case of EphA2-Sam, where it provides crucial intermolecular contacts with ML surfaces thus contributing to head-to-tail type Sam-Sam associations. However, the α5-helix can also support Sam-Sam tail-to-tail associations characterized by contacts in between α5 helices from diverse Sam units facing each-others in parallel or antiparallel orientations [84,85]. Primary sequences of C-terminal helices in EphA2-Sam, Ship2-Sam and Odin-Sam1 all include a proline residue and a few basic amino acids. In theory it could be possible to hamper Sam-Sam associations mediated by EphA2-Sam with negatively charged peptides targeting directly the receptor C-terminal α5-helix thus avoiding interactions with the negatively charged ML sites of Ship2-Sam and Odin-Sam1. However, in principle such negatively charged helical peptides could also target the C-terminal α5 helices of Ship2-Sam and Odin-Sam1 and allosterically interfere with formation of EphA2-Sam/Ship2-Sam and/or EphA2-Sam/Odin-Sam1 complexes. On the other side helical peptides with less negative charge could instead block access of EphA2-Sam to the ML sites of Odin-Sam1 and Ship2-Sam simulating head-to-tail Sam-Sam associations.

**Figure 12:**
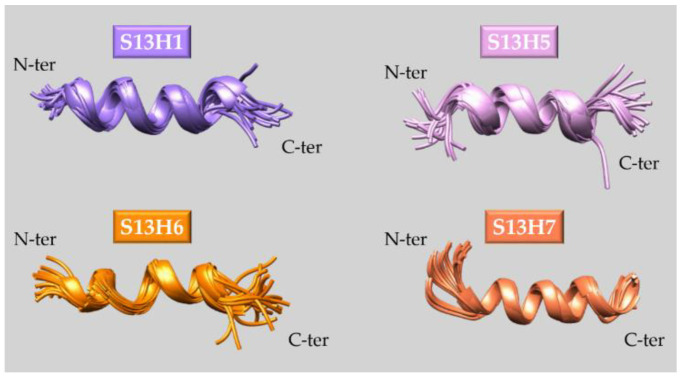
Helical peptides enriched in charged residues. NMR structures of S13H1, S13H5, S13H6 and S13H7 peptides: the twenty conformers forming each NMR ensemble are overlayed on the backbone atoms and shown in the ribbon representation [191].

With this in mind a few charged peptides enriched in amino acids with high helical propensities were designed (See peptides S13H1, S13H4, S13H5, S13H6 and S13H7 in Table 2) [191]. As helix starting point a Proline residue was inserted in the peptide sequences at the N-terminal side and was flanked by two negatively charged amino acids (an Aspartic and a Glutamic acid), the negatively charged residues could indeed stabilize the positive macrodipole of a potential helical arrangement; likewise, a positively charged amino acid was added towards the C-terminal side to further induce a “capping effect” and stabilize a negatively charged C-terminal helical macrodipole [204]. Nevertheless, to favour the helical conformation, opposite charged residues were inserted in the i and i+3 and/or i+4 positions to allow formation of salt bridges between acidic and basic amino acids side chains; finally, hydrophobic amino acids with high helical propensity (such as Alanine, Methionine, Leucine) [205] were introduced into the sequences as well (Table 2). The helical content in the designed sequences was predicted with the software Agadir and ranged from 36% (S13H1) to 64% (S13H5) [206]. Peptide conformational preferences were experimentally analysed through CD and NMR spectroscopies [191]. Detailed NMR studies revealed for S13H4 peptide a more disordered flexible conformation. An NMR structure could be calculated in PBS instead for S13H1, S13H5, S13H6 and S13H7 peptides revealing their helical structuration (Figure 12) [191]. Interestingly, CD spectra recorded at different peptide concentrations indicated as well a certain propensity of a few peptides, in particular S13H7, to aggregate and form possibly coiled-coil systems at high concentrations [191].

The stability of peptide helical conformations was analysed through MD simulations that showed for S13H1 a largely fluctuating helix while the most stable helical arrangement was detected for S13H6.

Interaction studies in between peptides and different Sam domains were conducted as well by a variety of techniques (NMR, SPR and MST) and pointed out S13H4 and S13H7 as weak binders of Odin-Sam1 (Table 3) [191].

The detailed structural analyses conducted for the designed linear helical peptides revealed their dynamic nature and let speculate the possible coexistence in solution of multiple species (i.e., ordered monomeric helical conformations, disordered forms, and to a lesser degree aggregated coiled-coil systems) and complex equilibria in which they might be involved. This feature could prevent high affinity interactions in between target Sam domains and helical peptides.

#### 5.3.2. Stapled Peptides

**Figure 13:**
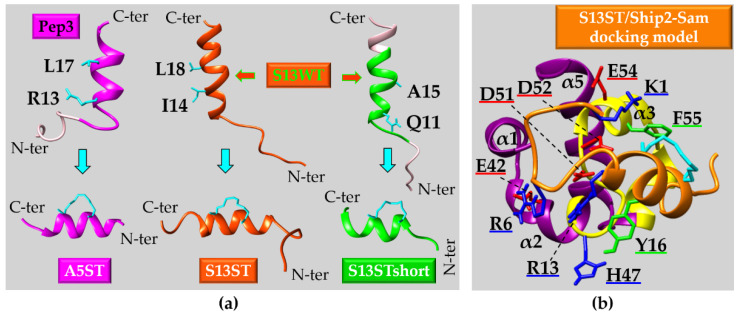
Design of stapled peptides. (**a**) The NMR structures of Pep3 and S13WT (conformers n.1) are reported on the top in a ribbon representation; the side chains of residues replaced in (S)-2-(4′-pentenyl)-alanines in the stapled peptides are shown in cyan. The NMR structures of diverse stapled peptides (A5ST (PDB entry 6F7O), S13ST (PDB entry 6F7M), and S13STshort (PDB entry 6F7N) [192]) are reported at the bottom with the hydrocarbon stapling bridge highlighted in cyan. Two different stapled peptides (i.e., S13ST and S13STshort) derive from S13WT peptide, whereas the A5ST stapled peptide derives from Pep3. The Pep3 and S13WT regions shown in pink represent those excluded from theA5ST and S13STshort sequences, respectively. (**b**) Best docking pose of the S13ST (orange)/Ship2-Sam (violet with the ML region colored yellow) complex. The side chains of a few peptide and protein residues performing intermolecular interactions are reported in red (negatively charged amino acids), blue (positively charged amino acids), green (hydrophobic amino acids). The docking model was generated with Autodock Vina (version 1.1.2) [208] starting from the NMR structures (i.e., first conformers) of Ship2-Sam and S13ST. The software ADT (AutoDock Tools) [209] was employed to convert the .pdb in .pdbqt files, during conversion the bonds connecting backbone atoms of S13ST residues in helical conformation (from K12 to L21) were set as non-rotatable. The search grid was centred on the Ship2-Sam ML interface (x center: 8.765 Å, y center: 8.152 Å, z center: 3.353 Å; x and y sizes: 40 Å, z size: 34 Å). The number of output models was set to 9. The analysis of intermolecular interactions in the output docking poses was performed with the ADT software.

The second strategy that was adopted to generate Sam domain targeting helical peptides was the hydrocarbon stapling technique [207]. The design of this kind of peptides started from Pep3 [188] and S13WT [189] sequences that were previously identified by protein dissection approaches: Pep3 [188] and S13WT [189] encompass the α5 C-terminal helices and adjacent regions of Odin-Sam1 and EphA2-Sam, respectively (Figure 9a and Figure 10 and Table 2). A helical S13WT peptide could bind the ML interface of Ship2-Sam by mimicking EphA2-Sam EH interface (see Section 5.1.2) [189], whereas a helical Pep3 peptide that had unexpectedly residual binding to EphA2-Sam, could target the C-terminal helix of the receptor through a tail-to-tail interaction model (see Section 5.1.1) [188]. To force a helical conformation, two (S)-2-(4′-pentenyl)-alanines were inserted within S13WT and Pep3 sequences at positions spaced 4 residues apart and their side chains were joined together with an olefinic bond [192]. A crucial point during the design of stapled peptides is to choose the right positions where to insert the modified pentenyl-alanine residues as, it is important to introduce mutations in the starting sequences without hampering crucial interactions with the target protein. In silico docking studies are supportive at this stage. The NMR structures of Pep3 and S13WT peptides calculated in TFE were employed to build speculative models of Pep3/EphA2-Sam and S13WT/Ship2-Sam complexes, and identify those peptide residues whose side chains could provide the lowest number of contacts with the target Sam domains and that consequently, represented the best candidates to be replaced by pentenyl-alanines [192]. In detail, A5ST peptide was obtained by inserting a stapling bridge between position 13 and 17 of Pep3 sequence that was also shortened by cutting away the N-terminal unstructured portion (Table 2 and Figure 13a). A5ST conformation was analysed by CD and NMR, which indicated an ordered helical arrangement in aqueous environment. Indeed, the NMR structure of A5ST in PBS consists of a helix encompassing almost the whole peptide sequence (Figure 13a) [192].

S13ST peptide derives by S13WT sequence after adding a stapling bridge between I14 and L18 (Figure 13a). The S13STshort peptide version was instead generated by cutting away disordered N- and C-terminal tails of S13WT and adding a stapling bridge between Q11 and A15 (Table 2 and Figure 13a). NMR and CD conformational studies indicated that to obtain the best helical arrangement, differently from A5ST, which was highly helical in an aqueous environment, S13ST needed a low amount of TFE (i.e., 25%). The S13ST NMR structure calculated in PBS/TFE (75/25 *v*/*v*) is made up of a defined C-terminal helix including the stapling bridge, and a disordered N-terminal tail (Figure 13a). S13STshort peptide resulted highly insoluble in PBS buffer, so NMR characterization could be conducted only starting by a concentrated peptide stock solution in DMSO (Dimethyl sulfoxide) that was diluted in a PBS/TFE (50/50 *v*/*v*) mixture. The resultant S13STshort NMR structure includes a helix encompassing most of the peptide sequence (Figure 13a) [192].

Binding studies with EphA2-Sam and the A5ST peptide failed to reveal a high affinity interaction. Best results could be obtained with S13ST as NMR-based direct and competition-type interaction assays pointed out some binding of the peptide to Ship2-Sam ML region [192]. SPR and MST techniques confirmed the occurrence of an interaction and a K_D_ value in the high micromolar range for the S13ST/Ship2-Sam complex (Table 3). Interestingly the increase of helicity in S13ST with respect to the parent S13WT sequence was able to somehow improve binding to EphA2-Sam. To obtain further structural information about S13ST/Ship2-Sam complex, docking calculations were performed by employing the NMR structures (first conformers) of S13ST and Ship2-Sam and the software Autodock Vina (Figure 13b) [208]. In the computational model the S13ST peptide faces the Ship2-Sam ML interface (Figure 13b) but with a different orientation with respect to that adopted by EphA2-Sam α5 helix in complex with Ship2-Sam and Odin-Sam1 (Figure 6e and Figure 7c). In the docking pose the N-terminal region of the peptide, including a disordered stretch, is mainly involved into contacts with α2 and α3 helices of Ship2-Sam ML site. The peptide/protein interface is stabilized by an H-bond between E42 on Ship2-Sam α2 helix and R6 in S13ST (Figure 13b). Among residues in close contacts there are several negatively charged amino acids from Ship2-Sam (D51, D52 from α2α3 loop, E54 from α3 helix) and positively charged amino acids from S13ST (K1, R6, R13) (Figure 13b). Aromatic residues also provide intermolecular contacts: F55 from Ship2-Sam α3 helix interacts with K1 from S13ST, and Y16 (S13ST) is in close contact with H47 (α2 helix in Ship2-Sam) (Figure 13b).

As mentioned before, the stapling is useful to improve peptide drug-likeness. S13ST enzymatic stabilities in PBS and mouse plasma were tested and compared with those corresponding to the linear parental S13WT peptide. Both peptides appeared to be highly susceptible to proteolytic degradation, likely due to basic residues contained within their sequences, but S13ST resulted indeed slightly more stable [192].

Combined analyses of structural and interaction data collected for the peptides A5ST and S13ST suggested that a completely rigid peptide helical arrangement is alone not enough to induce a stronger binding to a target Sam domain, and that a balance between order and disorder may be needed to inhibit specific Sam-Sam interactions.

### 5.4. Designing Anticancer Peptides through In Silico Methods

Today, in silico methods have become very popular for the design and optimization of anti-cancer peptides as, with respect to traditional experimental techniques, are less time and cost consuming. In particular, Virtual Screening (VS) allows to search among large libraries of molecules the most promising hits against a target protein, and select them for further experimental studies. VS is a technique considered complementary to experimental High Throughput Screening, and can be also implemented to analyse peptide libraries [210]. Several tools have been developed to support computational peptide-based drug-discovery, such as programs for the identification or prediction of “hot-spot” regions in protein-protein complexes to be targeted by peptides, structure-based or ligand-based drug design tools optimized for peptides, 3D peptide structure prediction or modelling instruments. Several databases of biological active peptides, including anti-cancer peptides, have also been settled, together with tools to predict peptide ADME (Absorption, Distribution, Metabolism, and Excretion) properties, or useful for peptide optimization [210].

Computational approaches have been largely employed to discover inhibitors of Sam-Sam interactions mediated by EphA2 receptor as well. In this context, docking studies have been implemented not only to hypothesize a topology of binding between a specific Sam domain and a peptide ligand, as described thus far, but also to identify putative interactors from ad hoc designed virtual peptide libraries. In particular, since the use of peptides as therapeutics is limited by their sensitivity to proteolytic cleavage, which reduces their half-life, in order to limit enzymatic cleavage, Sam-targeting peptide sequences were generated by including non-natural amino acids, such as D-amino acids or employing cyclization routes [210].

#### 5.4.1. Virtual Screening Strategies to Identify Linear Peptides Targeting EphA2-Sam

**Figure 14:**
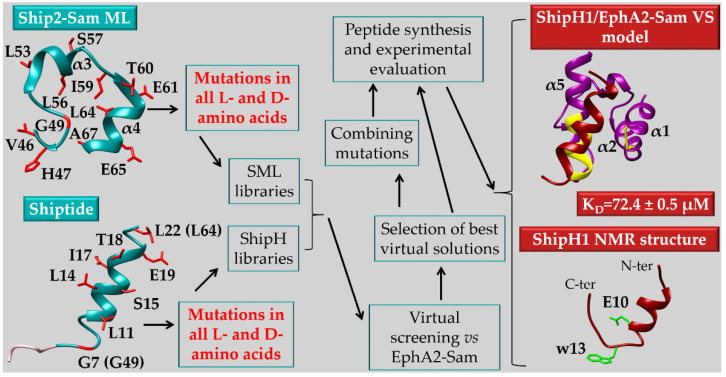
Virtual screening of peptide libraries modeled on Ship2-Sam ML interface. The 3D structures of Ship2-Sam ML region and of Shiptide peptide employed for peptide libraries design are shown, and the side-chains of residues, which were replaced with different amino acids, are colored red; Shiptide residue G7 and L22 correspond to G49 and L64, respectively, according to Ship2-Sam residue numbering (PDB entry 2K4P [68]). A synthetic workflow of the employed approach is also reported. On the right upper side, the docking model of the ShipH1/EphA2-Sam complex is shown with the EphA2-Sam EH site colored yellow; the dissociation constant measured experimentally for the complex is indicated. On the lower right side, the first conformer of the ShipH1 NMR structure calculated in presence of TFE is shown with the side chains of residues that are mutated with respect to the native Shiptide sequence highlighted in green [193].

The ML interface of Ship2-Sam represents a good starting point to generate virtual peptide libraries to be screened against the Sam domain of EphA2. First, two sets of virtual libraries of linear peptides (“SML” and “ShipH” peptide series (Figure 14a and Table 2)) were designed [193]. The 3D coordinates of the Ship2-Sam ML region (i.e., amino acid region from V46 to A67 following residue numbering of the PDB entry 2K4P [68]), which were extracted from the structure of the entire Sam domain, were employed as starting point to build SML libraries. Within the amino acid positions of the selected ML region, mutations with all diverse L- and D-amino acids were inserted at 12 ad hoc chosen sites that according to NMR and docking studies of the Ship2-Sam/EphA2-Sam complex [68,163] should have been less crucial for Sam-Sam association (Figure 14). This approach led in the end to 12 SML virtual peptide libraries, each containing the wild-type sequence and 38-point mutants with 3D structures similar to the one observed in the ML region of Ship2-Sam domain (Figure 14) [193].

Instead, ShipH libraries were generated by focusing on the Shiptide peptide (See Section 5.1.1) starting from the 3D coordinates of the Shiptide NMR structure calculated in H_2_O/TFE mixture (30/70 *v*/*v*) after cutting away the disordered N-terminal tail (Figure 14). In detail, the Ship2-Sam sequence from N48 to L64 positioned inside the ML interface (Figure 8a and Figure 14a) was employed for this second set of virtual libraries generation. Again, within the selected ML sequence, amino acid positions considered, according to docking studies, to be dispensable for the binding of the Shiptide to EphA2-Sam [187], were mutated in all diverse L- and D-amino acids. This second approach led to the creation of 8 virtual helical peptide libraries (Figure 14) [193].

Each peptide library was screened against the EH region of EphA2-Sam with Autodock-Vina [208]. From each library screening, the five best-ranked solutions underwent further filtering, in which sequences with redundant type of mutations were filtered away and those that could enhance the binding to EphA2-Sam, such as mutations in negatively charged or aromatic residues, were favored [193]. In the case of ShipH peptides, different mutations were even combined together, generating double-mutant peptides (ShipH1 and ShipH2 in Table 2) (Figure 14). At the end of computational procedure, 8 peptides (Sequences SML6-11, ShipH1,2 in Table 2) were chosen to be synthetized and experimentally tested, together with a peptide, named CTRL (Table 2), reproducing the wild-type Ship2-Sam sequence that was used for comparison purpose [193].

Experimental interaction studies performed via SPR, MST and NMR techniques pointed out ShipH1 peptide as a novel weak EphA2-Sam ligand (Table 2). This peptide is a double mutant containing an additional negative charge with respect to the wild-type corresponding sequence and a D-Tryptophan (Table 2). NMR direct and competition-type binding assays revealed that ShipH1 targets the EH region of EphA2-Sam and can be gradually displaced from the complex by Ship2-Sam [193]. Conformational studies, carried out by CD and NMR spectroscopies, indicated that ShipH1 peptide is highly disordered in aqueous environment. In fact, an NMR structure of the peptide, represented by an ordered helical conformation encompassing the N-terminal region and a disordered C-terminal tail, could be calculated only in presence of 80% TFE (Figure 14). Interestingly, ShipH1 is characterized by an improved serum stability with respect to the wild type peptide (CTRL in Table 2), likely due to the presence within its sequence of a D-amino acid [193].

#### 5.4.2. Cyclic Peptide Libraries against EphA2-Sam

**Figure 15:**
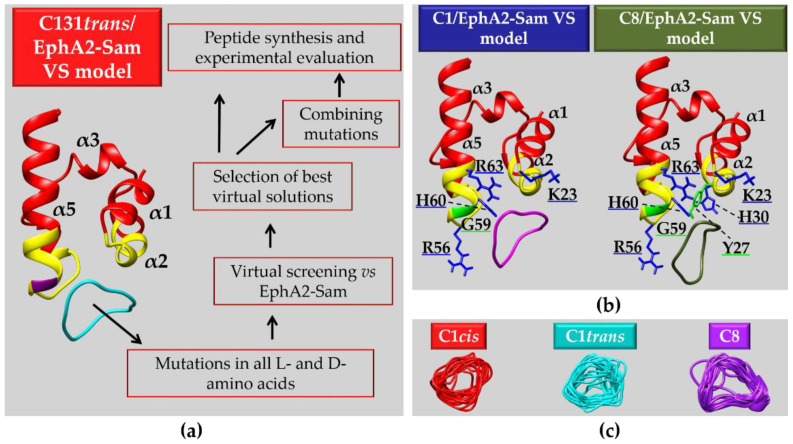
(**a**) Design of cyclic peptide libraries and virtual screening approach. On the left side the model of the C131*trans*/EphA2-Sam complex, obtained by Autodock Vina, is shown, with the targeted EphA2-Sam region colored yellow with G59 evidenced in magenta. (**b**) Virtual screening results for C1/EphA2-Sam and C8/EphA2-Sam complexes. A few crucial EphA2-Sam residues providing intermolecular interactions are colored in blue (positively charged side chains) and green (hydrophobic side chains). (**c**) NMR structures of C1*cis*, C1*trans* (calculated in H_2_O) and C8 (calculated in PBS/TFE with ~69% TFE): twenty conformers are superimposed on the backbone atoms.

As already described in Section 4.1.1, in the EphA2-Sam/Ship2-Sam complex a hydrogen bond between the backbone H_N_ of G59 (sequence numbering according to the PDB entry 2E8N), positioned at the N-terminal side of the α5 C-terminal helix of EphA2-Sam, and the backbone _C_O of N48 (sequence numbering according to the PDB entry 2K4P), located on the α2 helix of Ship2-Sam, represents possibly an anchoring point for Sam-Sam associations of the ML/EH type [89,92]. Consequently, blocking formation of this H-bond through a peptide ligand could be an efficient route to inhibit the binding of EphA2-Sam to partners Sam domains. The EphA2-Sam region positioned around G59 includes the N-terminal side of α5 C-terminal helix, the α4α5 loop and the α2 helix, and contains several positively charged and aromatic residues (Figure 15). Thus, the amino acid sequence “YEAGENFPNEGAE”, somehow complementary to the target EphA2-Sam region was first conceived. This peptide sequence contains a few negatively charged and aromatic residues, along with polar amino acids, which could eventually provide additional H-bond interactions, and a Proline residue that should favor the formation of turn-like structures and support cyclization. Head-to-tail cyclization of this sequence through a lactame bond led to the C131 peptide (Table 2) [92]. The 3D structure of the cyclic C131 peptide was built by molecular modelling with UCSF Chimera [177] by setting the Proline residue in the *trans* and *cis* configurations. Docking studies with Autodock Vina [208] let speculate that indeed the C131*trans* cyclic organization could well target the selected EphA2-Sam region (Figure 15a). Thus, design of cyclic peptide libraries was focused on C131*trans* (Table 2); in detail, to generate diverse peptide libraries, every residue within C131 primary sequence was mutated in all L- and D- amino acids, the head-to-tail cyclic backbone arrangement and the *trans* Proline configurations was set in each library member. A total of 13 libraries came out from this approach, and they were virtually screened against EphA2-Sam. In the end, the 10 best ranked solutions from each library screening were analyzed, and among them a few sequences (i.e., C4, A5, C1, C5, C6 in Table 2) provided with non-conservative mutations with respect to the starting C131 sequence were synthetized and experimentally validated to explore peptides having diverse chemical features [92]. Additional double and triple peptide mutants were obtained by combining best scoring point mutations together (See B2, C7, C8 and C9 sequences in Table 2) [92], and next synthetized and experimentally analyzed [92].

Experimental binding studies by NMR and SPR techniques indicated that most of the selected peptides were unable to relevantly bind EphA2-Sam. Indeed, only C1 and C8 peptides induced a signal variation in SPR experiments when injected at 400 µM concentration as analytes while keeping EphA2 immobilized on the microchip surface [92]. The inspection of docking poses of C1/EphA2-Sam and C8/EphA2-Sam complexes indicated that peptide residues could interact with crucial positively charged and aromatic residues comprised within the targeted protein region (Figure 15b). Thus, further SPR assays were conducted with C1 and C8 in a wide concentration range but failed to provide a reliable K_D_ value for the cyclic peptide/EphA2-Sam complexes as no dose-response variation could be revealed for C1, whereas no saturation condition was reached for C8 that also gave a large unspecific signal [92].

To gain further information useful to better explain negative results from experimental interaction studies, deep structural analyses of C1 and C8 were conducted by CD and NMR techniques [92]. NMR spectra of C1 peptide were registered in H_2_O and revealed the occurrence of two set of signals, ascribable to Proline *cis-trans* isomerization. NMR structures of C1 were calculated with both proline in *cis* and in *trans* configurations concluding, in agreement with CD data, that C1 peptide was provided with a flexible cyclic arrangement lacking specific secondary structure elements [92] (Figure 15c).

Concerning the C8 peptide, NMR conformational studies were first conducted in aqueous environment and indicated the Proline residue in *trans* configuration but still a flexible/disordered cyclic arrangement. Further NMR structural studies were carried out for C8 in presence of TFE that, according to analyses of CD spectra, was able to induce a change in the H-bond network of the peptide [92]. Although NMR studies showed a partial increase of peptide rigidity induced by TFE and again the occurrence of Proline in only *trans* configuration, they also pointed out that the peptide lacked canonical secondary structure elements and that could sample multiple conformations (Figure 15c) [92].

Conformational analyses performed for other cyclic peptides revealed structural flexibility, and in most cases the coexistence in solution of both *cis* and *trans* Proline isomers [92]. Thus, structural analyses of this set of cyclic peptides suggested a possible relationship between failure to bind EphA2-Sam and conformational flexibility.

## 6. Conclusions and Future Perspectives

The Sam domain of EphA2 receptor can be considered an innovative target to be explored in anticancer drug discovery [63]. EphA2 is over-expressed in many tumors, and the process of ligand induced receptor endocytosis and the consequent degradation are under investigation as ways to decrease tumor malignancy. In this context, the heterotypic Sam-Sam interactions of EphA2 with the lipid phosphatase Ship2 and the adaptor protein Odin, by modulating receptor endocytosis and stability [166,167], hold a certain interest as target PPIs to discover novel therapeutic compounds. Indeed, during the last years we focused our research activity to identify peptides able to hamper Sam-Sam associations involving EphA2 and eventually induce receptor endocytosis and degradation thus lowering pro-oncogenic signaling pathways.

Ship2-Sam/EphA2-Sam and Odin-Sam1/EphA2-Sam complexes are characterized by the well-known ML/EH interaction model, stabilized mainly by electrostatic contacts and whose structural features have been largely elucidated [68,70]. Based on structural data, protein dissection approaches were first explored to check if isolated peptide sequences encompassing ML and EH sites could preserve ability to bind partner Sam domains [187,188,189]. This approach provided weak EphA2-Sam interactors like the Shiptide and Pep2 modeled on the ML interfaces of Ship2-Sam [187] and Odin-Sam1 [188], respectively (Table 2 and Table 3). Detailed structural and biophysical studies let speculate that such linear peptides lacking a native like fold could be too disordered to achieve efficient Sam domains binding as further confirmed by analyses of peptides mimicking the EH site of EphA2-Sam (i.e., S13WT and S13SS in Table 2) [189].

To overcome the issue, peptides having more rigid helical folds such as linear peptides provided with helix promoting residues [191] and hydrocarbon stapled peptides [192] were designed and analyzed. Through these other strategies the Odin-Sam1 targeting peptide S13H7 was identified [191] along with the stapled peptide S13ST reproducing the α5 helix of EphA2-Sam and able to interact with Ship2-Sam better than the linear analogue S13WT (Table 2 and Table 3) [192]. However, even peptides with more organized helical conformations failed to bind Sam domains with affinities comparable to those characterizing the Sam-Sam interactions to be targeted. Results from these studies indicated that a fine tuning between conformational rigidity and flexibility was possibly needed to achieve a robust interaction [191,192].

Further work on cyclic peptides confirmed this hypothesis [193]. In fact, the introduction of a Proline residue within peptide sequences to favor head-to-tail backbone cyclization, added a conformational variability that overcame the complementary in amino acids types between EphA2-Sam target region and designed cyclic peptide sequences (Table 3) [193]. Again, detailed conformational analyses coupled with interaction studies demonstrated that a flexible cyclic arrangement able to assume multiple conformational states, such as those related to *cis-trans* Proline isomerization, was unable to efficiently target Sam domains and suggested that much attention needed to be given to cycle length and amino acid types to properly modulate flexibility [193].

Nevertheless, by analyzing EphA2-Sam sequence in and around the EH interface, the “KRIAY” motif was identified and the linear KRI3 peptide designed (Table 3) [189]. KRI3 demonstrated to be a weak Ship2-Sam ligand and antagonist of its association with EphA2-Sam (Table 2 and Table 3) [189]. In addition, KRI3 also presented some cytotoxicity towards PC3 prostate cancer cells and even in absence of conjugation to a CPP (Cell Penetrating Peptide) sequence was able to penetrate cells [189,190]. Data collected on KRI3 and a few derived peptides indicated that the largest challenge in designing KRI3 analogues was to avoid aggregation and unspecific electrostatic interactions due to the large content of positively charged residues [189,190]. In the close future we can envision analyzing KRI3 modified peptides provided with unnatural amino acids and exploring alternative cyclization routes to improve peptide stability.

It’s worth noting that, when dealing with protein-protein interactions mediated by electrostatic contacts, as in the cases of the EphA2-Sam/Ship2-Sam and EphA2-Sam/Odin-Sam1 complexes, it is challenging to avoid unspecific interactions at sites different from the binding loci; for example, Ship2-Sam possesses, along with the negatively charged ML interface, another region dense in acidic residues between helices α1 and α2, as often mentioned in our studies [189,190].

Another aspect that we have not yet well investigated is the selectivity of our peptides towards diverse Sam domain mediated interactions driven by electrostatic contacts. In order to address this point, the panel of proteins under investigation needs to be enlarged beyond the three simple set EphA2-Sam, Ship2-Sam, Odin-Sam1.

The large amount of collected data and the multeplicity of implemented strategies reflect the challenges related to target Sam domains lacking deep pockets and/or clefts where to allocate compound inhibitors. In the end thus far we have identified several weak Sam targeting peptides that represent ideal “starting material” to be optimized concerning binding affinity and selectivity.

## Figures and Tables

**Table 1:** List of a few peptide-based drugs and diagnostic tools approved by FDA from 2019 to 2022. Peptides for anti-cancer treatments and cancer diagnosis are highlighted in the gray rows.

Name	Target Disease or Application Field	Year of Approval
Scenesse^®^	Erythropoietic protoporphyria	2019 [52,53]
^68^Ga-DOTATOC	Positron Emission Tomography (PET) for localization of somatostatin receptor-positive NEuroendocrine Tumors (NETs) in adult and pediatric patients	2019 [52,53]
Vyleesi^®^	Hypoactive sexual desire disorder in premenopausal women	2019 [52,53,54]
^68^Ga-PSMA-11	PET imaging of Prostate-Specific Membrane Antigen (PSMA)-positive lesions in men affected by prostate cancer	2020 [55,56]
Imcivree^®^	Chronic weight management in adults and pediatric patients (age ≥ 6 years old) with obesity	2020 [54,55,56]
Detectnet^TM^	PET for localization of somatostatin receptor-positive NETs in adult patients	2020 [55,56]
Sogroya^®^	Replacement therapy for growth hormone deficiency in adults	2020 [56]
Voxzogo^®^	Achondroplasia in pediatric patients who are 5 years of ageand older with open epiphyses	2021 [57,58]
Korsuva^TM^	Moderate-to-severe pruritus linked to chronic kidney disease in adults undertaking hemodialysis	2021 [57,58]
Bylvay^®^	Pruritus in patients (age ≥ 3 months old) affected by progressive familial intrahepatic cholestasis	2021 [57,58]
Pylarify^®^	PET imaging of PSMA-positive tumors in men with prostate cancer	2021 [57,58]
Empaveli^®^	Paroxysmal nocturnal hemoglobinuria in adults	2021 [57,58]
Zegalogue^®^	Severe hypoglycemia in patients (age ≥ 6 years old) with diabetes	2021 [57,58]
Pepaxto^®^	Relapsed or refractory multiple myeloma in adult patients	2021 [57,58]
Lupkynis^®^	Lupus nephritis in adults	2021 [54,57,58]
Mounjaro^TM^	Improvement of glycemic control in adults with type 2 diabetes mellitus, used in combination with diet and exercise	2022 [59,60]

**Table 2:** List of diverse strategies employed to design potential EphA2-Sam/Ship2-Sam or EphA2-Sam/Odin-Sam1 inhibitors with related peptide names and sequences. Ac- and NH_2_- indicate acetyl N-terminal and amide C-terminal protections, respectively. Sequence mutations with respect to the reference peptides KRI3, CTRL and C131 are shown in green, red and blue respectively. In stapled peptides sequences (X) stands for (S)-2-(4′-pentenyl)-alanine while, in peptides from computational screening approaches D-amino acids are reported with lower case letters.

	Peptide Name	Peptide Sequence
**Ship2-Sam** **dissection**	Shiptide	Ac-EGLVHNGWDDLEFLSDITEEDL-NH_2_
**Odin-Sam1** **dissection**	Pep1	Ac-SKLLLNGFDDVHFLGSNVMEEQ-NH_2_
	Pep2	Ac-SKLLLNGFDDVHFLGSNVMEEQ DLRDIGISDPQHRRKLLQAAR-NH_2_
	Pep3	Ac-DLRDIGISDPQHRRKLLQAAR-NH_2_
**EphA2-Sam** **dissection**	S13WT	Ac-KRIGVRLPGHQKRIAYSLLGLKDQV-NH_2_
	S13-SS	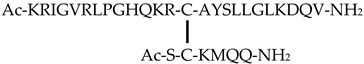
	KRI	Ac-GHQKRIAY-NH_2_
	KRI2	Ac-KRIAYKRIAY-NH_2_
	KRI3	Ac-KRIAYKRIAYKRIAY-NH_2_
**KRI3** **analogues**	KRI3-YM	Ac-KRIAAKRIAAKRIAA-NH_2_
	KRI3-IM	Ac-KRKAYKRKAYKRKAY-NH_2_
	KRI4	Ac-KRIAYKRIAYKRIAYKRIAY-NH_2_
	cKRI3	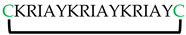
**Helical peptides enriched in** **charged residues**	S13H1	Ac-DPETEEIAYKLAMLKAQ-NH_2_
	S13H4	Ac-DPETKRIAYKLAMLKAQ-NH_2_
	S13H5	Ac-DPETEEIAKRLAMLAQK-NH_2_
	S13H6	Ac-DPETKRIAEELAMLAQK-NH_2_
	S13H7	Ac-DPETEEIAWILAMLAQK-NH_2_
**Stapled peptides**	A5ST	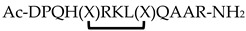
	S13ST	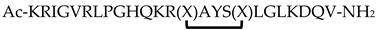
	S13STshort	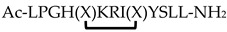
**Peptides from computational screening** **approaches**	SML6	Ac-VHNGWDDLEFfSDITEEDLEEA-NH_2_
	SML7	Ac-VENGWDDLEFLSDITEEDLEEA-NH_2_
	SML8	Ac-VHNGWDDLEFWSDITEEDLEEA-NH_2_
	SML9	Ac-VHNyWDDLEFLSDITEEDLEEA-NH_2_
	SML10	Ac-VHNGWDDLEFQSDITEEDLEEA-NH_2_
	SML11	Ac-VHNGWDDLEFLSDITEEDLnEA-NH_2_
	ShipH1	Ac-NGWDDLEFLEDIwEEDL-NH_2_
	ShipH2	Ac-NGWDDnEFdSDITEEDL-NH_2_
	CTRL	Ac-EGLVHNGWDDLEFLSDITEEDLEEA-NH_2_
	C131	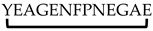
	C4	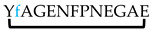
	A5	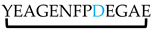
	C1	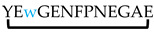
	C5	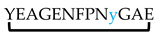
	C6	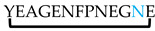
	B2	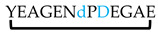
	C7	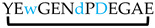
	C8	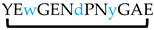
	C9	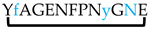

**Table 3:** List of dissociation constants (K_D_) obtained by SPR and/or MST techniques for peptide ligands of target Sam domains.

Peptide/Protein Complex	K_D_ (µM)
Pep2/EphA2-Sam	221.97 ± 0.01 (SPR) [188]
Pep3/EphA2-Sam	523.11 ± 0.01 (SPR) [188]
KRI3/Ship2-Sam	83 ± 8 (SPR) [189]
KRI3-IM/Ship2-Sam	309 ± 4 (MST) [190]
cKRI3/Ship2-Sam	140 ± 20 (SPR) [190] 73 ± 5 (MST) [190]
S13H4/Odin-Sam1	320 (SPR) [191]
S13H7/Odin-Sam1	62 ± 3 (SPR) [191] 68 ± 3 (MST)
S13ST/Ship2-Sam	52.2 ± 0.7 (MST) [192]
ShipH1/EphA2-Sam	72.4 ± 0.5 (MST) [193]
